# CD44 Receptor Mediates Urate Crystal Phagocytosis by Macrophages and Regulates Inflammation in A Murine Peritoneal Model of Acute Gout

**DOI:** 10.1038/s41598-020-62727-z

**Published:** 2020-04-01

**Authors:** Emira Bousoik, Marwa Qadri, Khaled A. Elsaid

**Affiliations:** 1 Department of Biomedical and Pharmaceutical Sciences, Chapman University School of Pharmacy, Chapman University, Irvine, CA USA; 2 School of Pharmacy, Omar-Al-Mukhtar University, Derna, Libya; 3 Department of Pharmacology, College of Pharmacy, Jazan University, Jazan, 82826 Saudi Arabia

**Keywords:** Acute inflammation, Gout

## Abstract

Gout is a chronic arthritis caused by the deposition of poorly soluble monosodium urate monohydrate (MSU) crystals in peripheral joints. Resident macrophages initiate inflammation in response to MSU mediated by NF-κB nuclear translocation and NLRP3 inflammasome activation. We investigated the role of CD44, a transmembrane receptor, in mediating MSU phagocytosis by macrophages. We used an antibody that sheds the extracellular domain (ECD) of CD44 to study the role of the receptor and its associated protein phosphatase 2A (PP2A) in macrophage activation. We also studied the significance of CD44 in mediating MSU inflammation *in-vivo*. *Cd44*^*−/−*^ BMDMs showed reduced MSU phagocytosis, LDH release, IL-1β expression and production compared to *Cd44*^*+/+*^ BMDMs. Elevated CD44 staining was detected intracellularly and CD44 colocalized with α-tubulin as a result of MSU exposure and ECD-shedding reduced MSU phagocytosis in murine and human macrophages. Anti-CD44 antibody treatment reduced NF-κB p65 subunit nuclear levels, IL-1β expression, pro-IL-1β and IL-8 production in MSU stimulated THP-1 macrophages (*p* < *0.01*). The effect of the antibody was mediated by an enhancement in PP2A activity. CD44 ECD-shedding reduced the conversion of procaspase-1 to active caspase-1, caspase-1 activity and resultant generation of mature IL-1β in macrophages. Neutrophil and monocyte influx and upregulated production of IL-1β was evident in wildtype mice. MSU failed to trigger neutrophil and monocyte recruitment in *Cd44*^*−/−*^ mice and lower IL-1β levels were detected in peritoneal lavages from *Cd44*^*−/−*^ mice (*p* < *0.01*). Anti-CD44 antibody treatment reduced neutrophil and monocyte recruitment and resulted in reduced lavage IL-1β levels in the same model. CD44 plays a biologically significant role in mediating phagocytosis of MSU and downstream inflammation and is a novel target in gout treatment.

## Introduction

Gout is a chronic arthritic condition characterized by intermittent episodes of acute flares of pain and inflammation that commonly occurs in peripheral synovial diarthrodial joints due to the deposition of poorly soluble sodium urate crystals^1–5^. Gout sits at the intersection of a number of chronic inflammatory and metabolic diseases and is associated with increased incidence of type-2 diabetes, hypertension, hyperlipidemia, chronic kidney disease and osteoarthritis^6–10^. The prevalence of gout has been steadily increasing and refractory, difficult-to-treat gout has been reported in more than 1% of all patients and is characterized by the presence of ongoing active disease despite the use of anti-inflammatory and urate lowering therapy^11,12^. Several studies show that inadequate therapeutic management of acute gout is prevalent and continues to grow^13–16^. Considering the severe toxicities reported with high-dose colchicine and other existing anti-inflammatory drugs, there is a significant need to identify novel pharmacologic targets with the goal of controlling inflammation in acute gout^17–19^.

In response to urate crystals, joint resident macrophages initiate inflammation through crystal phagocytosis and production of interleukin-1 beta (IL-1β) and other chemokines^5,20,21^. The exact mechanism of crystal phagocytosis by joint macrophages is not entirely understood. The toll-like receptors (TLRs) family, particularly TLR2 and TLR4, were thought to mediate phagocytosis of urate crystals as part of an innate immune response in the joint^22^. TLR2 and TLR4-deficient macrophages exhibited reduced phagocytosis of urate crystals and downstream IL-1β production^22^. However, in a subsequent study, the finding that TLR1 through 9-deficient mice lacked an inflammatory response to MSU crystals *in vivo* challenged the role of TLRs in gout pathogenesis^23^. In a recent study, we have shown that recombinant human proteoglycan-4 (rhPRG4) reduced urate crystal phagocytosis by macrophages, inhibited nuclear factor kappa b (NF-κB) pathway, NOD-, LRR- and pyrin domain-containing protein 3 (NLRP3) inflammasome activation, and suppressed IL-1β expression and secretion^24^. PRG4 is a mucinous glycoprotein that is a major component of synovial fluid, where it fulfills lubricating and joint homeostatic roles^25^. PRG4 was recently identified as a cluster determinant-44 (CD44) ligand where it has an anti-inflammatory role in the joint and an ability to regulate TLR2 and TLR4 receptor stimulation^26–29^. The contribution of CD44 versus TLR2 or TLR4 receptors to the observed efficacy of rhPRG4 in preventing urate crystal phagocytosis by macrophages was further studied^24^. PRG4 was shown to bind preferentially to CD44 compared to TLR2 or TLR4 receptors on macrophages, which may suggest that CD44 is implicated in the phagocytosis of urate crystals by macrophages^24^.

CD44 is a highly glycosylated transmembrane receptor with various isoforms generated by extensive alternative splicing and posttranslational modifications, and is widely expressed in immune and connective tissue cells^30^. CD44 contributes to the reception of a broad array of microenvironmental signals including its own ligands, cytokines and growth factors^31^. CD44 functions to regulate the activation of TLRs^32–34^. In a recent study, we have shown that with using either a CD44 ligand, *e.g*. hyaluronic acid or an antibody that induces the shedding of CD44 extracellular domain (ECD), agonist-induced TLR2 activation was suppressed in macrophages^34^. HA-induced CD44 receptor internalization or antibody-mediated ECD shedding reduced NF-κB nuclear translocation leading to attenuation of tumor necrosis factor alpha (TNF-α) expression^34^. Engagement of CD44 by the antibody resulted in protein phosphatase-2 A (PP2A) activation, and inhibition of PP2A activity reversed the anti-inflammatory effect of the antibody^34^.

The aim of this investigation was to evaluate the singular role of CD44 in facilitating urate crystal phagocytosis by macrophages and study its function in regulating downstream NF-κB nuclear translocation and NLRP3 inflammasome activation and IL-1β production *in vitro* and *in vivo* using a peritoneal model of acute gout. We hypothesized that CD44 mediates the phagocytosis of urate crystals by macrophages and CD44 receptor neutralization and/or deficiency reduces crystal phagocytosis, NF-κB translocation and NLRP3 inflammasome activation and suppresses urate crystal-linked inflammation.

## Materials and Methods

### Generation of bone marrow derived macrophages (BMDMs) from *Cd44*^*+/*+^ and *Cd44*^*−/−*^ mice and study of the phagocytosis of latex beads and urate crystals by BMDMs, crystal-induced cytotoxicity, IL-1β expression and production studies

*Cd44*^*+/+*^ (JAX stock # 00664) and *Cd44*^*−/−*^ (JAX stock # 005085) pathogen-free male mice (n = 15 in each group) were acquired from the Jackson Laboratory (Maine, USA)^35^. Animals (12–14 weeks old) were euthanized under CO_2_ and isolation of bone marrows and differentiation into BMDMs were performed as described^34,36^. Generation of BMDMs was confirmed using flow cytometry^34^. All animal experiments were approved by the IACUC committee at Chapman University. All experiments were performed in accordance with all applicable guidelines and regulations.

Phagocytic activity of BMDMs was determined using a Phagocytosis Activity Assay Kit (IgG FITC) (Cayman Chemicals). Briefly, *Cd44*^*+/+*^ and *Cd44*^*−/−*^ BMDMs were seeded in 6 well plates (500,000 cells per well) and allowed to adhere overnight. Latex bead rabbit IgG -FITC complex was added to the culture medium at a 1:100 dilution and incubated at 37 °C for four hours. Cells were washed twice with the assay buffer. To distinguish cells that have internalized the beads from those binding the beads at the surface, cells were incubated with a trypan blue quenching solution for two minutes. Cells were then washed twice with the assay buffer. Finally, cells were gently scraped and cell-associated fluorescence intensities were determined using flow cytometry.

Urate crystal phagocytosis by *Cd44*^*+/+*^ and *Cd44*^*−/−*^ BMDMs was performed by seeding BMDMs onto coverslips in 6 well plates (500,000 cells per well) followed by incubation with pyrogen-free monosodium urate monohydrate (MSU) crystals (100 μg/mL; Invivogen) for 4 hours at 37 °C. Subsequently, cells were fixed using 4% paraformaldehyde followed by washing with PBS. Following permeabilization with 0.1% Triton X-100 and washing with PBS, fluoroshield-mounting medium with DAPI (Abcam) was added and incubated over night at room temperature. BMDMs were visualized under the microscope and a blinded investigator determined the percentage of cells from each genotype that had intracellular MSU crystals. A total of at least 100 cells over 4–5 fields were examined across five independent experiments.

To investigate the impact of MSU crystals on the viability of BMDMs, we determined lactate dehydrogenase (LDH) activity levels in culture supernatants using an LDH Cytotoxicity Assay Kit (Abcam). BMDMs from both genotypes (500,000 cells per well) were treated with MSU crystals (100 μg/mL) for 1 and 4 hours followed by collection of culture supernatants, centrifugation at 600 × *g* for 10 min and determination of LDH activity.

To study the stimulation of *Cd44*^*+/+*^ and *Cd44*^*−/−*^ BMDMs by MSU crystals or lipopolysaccharide (LPS; Invivogen), cells were seeded in 6 well plates (500,000 cells per well) and treated with MSU (100 μg/mL) or LPS (100 ng/mL) for 6 and 72 hours, respectively. Total RNA extraction, quantification, cDNA synthesis and quantitative PCR (qPCR) were performed as previously described^34^. Genes of interest included IL-1β (Mm00434228_m1; ThermoFisher Scientific). The cycle threshold (Ct) value of IL-1β was normalized to the Ct value of GAPDH (Mm99999915_g1; ThermoFisher Scientific) in the same sample, and relative expression was calculated using the 2^−ΔΔCt^ method^37^. Data are presented as fold target gene expression in MSU treated macrophages relative to untreated control. IL-1β media concentrations in MSU or LPS treated macrophages were measured using an ELISA kit (R&D Systems).

### Differentiation of human THP-1 monocytes into macrophages and studies of MSU uptake by THP-1 macrophages or MCF-7 cells, time-dependent CD44 expression and receptor internalization

THP-1 monocytes (ATCC, USA) were differentiated into macrophages by incubation with phorbol 12-myristate-13-acetate (PMA; Sigma Aldrich) to a final concentration of 5 ng/mL for 48 hours^38^. Subsequently, media supernatants were removed and wells were washed with sterile PBS to remove any unattached cells and new RPMI 1640 media was added.

To further validate MSU phagocytosis by THP-1 macrophages, we compared MSU crystal uptake by THP-1 macrophages and the non-phagocytic MCF-7 breast carcinoma cells (ATCC)^39^. THP-1 and MCF-7 cells were seeded in 4-well chamber slides (250,000 cells per well; ThermoFisher Scientific) and were stimulated with MSU crystals (100 μg/mL) for 4 hours at 37 °C followed by washing with PBS. Direct visualization of MSU crystals under the microscope was performed as described above. Indirect assessment of MSU phagocytosis was determined by analyzing the change in cell side-scatter distribution due to crystal phagocytosis using a flow cytometer (BD FACSVerse) as described^24^. Two regions of interest were identified; P1 representing the macrophage population in the absence of MSU exposure and P2 representing the macrophage population with increased side scatter due to MSU phagocytosis. MSU-positive cells were calculated as the ratio of cells in the P2 region to the sum of cells in the P1 and P2 regions. This indirect method of determining MSU phagocytosis was previously validated by our group against the direct method of intracellular crystal visualization^24^. MSU crystal phagocytosis by THP-1 macrophages was also recorded over a 4-hour incubation period.

Time-dependent changes in CD44 expression and soluble CD44 media concentrations were determined following treatment of THP-1 macrophages with MSU crystals. THP-1 macrophages (500,000 cells per well) were incubated with MSU crystals (100 μg/mL) for 3, 6, 12 and 24 hours followed by media collections and cell harvest to perform CD44 gene expression studies as described above using commercially available primers and probes for CD44 (Hs01075864_m1) and GAPDH (Hs03929097_g1) (ThermoFisher Scientific). In another set of experiments, THP-1 macrophages were incubated with MSU crystals as described above followed by cell harvest and determination of cell-surface CD44 receptor density. THP-1 macrophages were incubated with an anti-CD44 antibody (Abcam) (1 μg per 1 × 10^6^ cells) for 1 hour at 4 °C. Following cell pelleting and washing with PBS, cells were subsequently incubated with DyLight^®^ 488 goat anti-mouse IgG at 1:200 dilution for 30 min at 4 °C. Following cell pelleting and washing, 4% paraformaldehyde (500 μL) was added and cell-associated fluorescence was determined by flow cytometry. Soluble CD44 receptor concentrations were determined using an ELISA kit (R&D Systems).

The internalization of CD44 following incubation of THP-1 macrophages with MSU crystals was determined using confocal microscopy and intracellular CD44 fluorescence intensity was quantified. THP-1 macrophages were seeded in 4-well chamber slides (ThermoFisher Scientific) followed by incubation with MSU crystals (100 μg/mL) for 3 or 6 hours. Subsequently, cells were fixed using 4% paraformaldehyde for 10 min followed by washing with PBS and permeabilization with 0.1% Triton X-100. Following washing with PBS, cells were blocked for 30 min in 2% bovine serum albumin (BSA) in PBS. Cells were incubated with anti-CD44 antibody (1:500 in 2% BSA; Abcam) overnight at 4 °C. Following washing with PBS, FITC-conjugated secondary antibody (1:1,000; Abcam) and Alexa Fluor 647-conjugated anti-α tubulin monoclonal antibody (1:1,000; Abcam) were added for 2 hours. Nuclear staining was performed using DAPI. Confocal imaging, Z-stacking and colocalization of CD44 and α-tubulin were performed as described^24^. At least 100 cells were examined in each group across 3 independent experiments and data are presented as the percentage of CD44 and α-tubulin-colocalization-positive cells. Intracellular mean CD44 fluorescence was also determined using at least 4 different fields in each group across 3 independent experiments and data are presented as fold change in intracellular CD44 fluorescence at 3 and 6 hours relative to control.

### Impact of anti-CD44 antibody treatment on the phagocytosis of MSU crystals by murine and human macrophages and downstream production of interleukin-1 beta (IL-1β) and interleukin-8 (IL8)

We used the IM7 clone as our anti-CD44 antibody in all *in vitro* and *in vivo* experiments^40^. IM7 is a monoclonal rat anti-CD44 antibody (ThermoFisher Scientific) that recognizes the ECD of all CD44 isoforms in murine and human cells and results in ECD shedding^40,41^. The mechanism of ECD shedding involves the activation of a sheddase enzyme, mediated by Rho GTPase activation and actin reorganization^41^. IM7 and rat isotype control (IC) antibodies (Abcam) were used at 2 μg/mL in all *in vitro* experiments. Murine J774A.1 macrophages (500,000 cells per well; ATCC) were seeded onto cover slips in 6 wells plates and were treated with MSU crystals (100 μg/mL) ± anti-CD44 or IC antibodies for 4 hours followed by direct determination of MSU crystals-positive cells as described above. We have also studied the impact of anti-CD44 antibody treatment on the phagocytosis of MSU crystals by THP-1 macrophages. THP-1 macrophages (250,000 cells per well) were incubated with MSU crystals (100 μg/mL) ± anti-CD44 or IC antibodies for 4 hours followed by analyzing the change in macrophage side scatter distribution as described above.

In a separate set of experiments, THP-1 macrophages were treated with MSU crystals (100 μg/mL) ± anti-CD44 or IC antibodies for 6 hours followed by media collections and cell harvest to perform IL-1β and IL8 gene expression studies as described above using commercially available primers and probes for IL-1β (Hs01555410_m1) and IL8 (Hs00174103_m1) (ThermoFisher Scientific). IL-1β and IL8 media concentrations were determined using ELISA kits (R&D Systems). Soluble CD44 receptor concentrations following treatments with anti-CD44 or IC antibodies were determined as described above.

### Analysis of NF-κB p65 subunit nuclear levels, NLRP3 inflammasome, pro-IL-1β, mature IL-1β, and caspase-1 activity following MSU challenge and impact of anti-CD44 antibody treatment

Quantification of the NF-κB p65 subunit nuclear levels was performed as previously described^34^. THP-1 macrophages were treated with MSU crystals (100 μg/mL) ± anti-CD44 or IC antibodies for 1 hour followed by cell harvest and extraction of nuclear protein (ThermoFisher Scientific). Nuclear levels of the p65 subunit were determined using a DNA binding ELISA assay (Abcam) and normalized to total nuclear protein content assayed using the micro BCA assay and expressed as detectable NF-κB p65 levels normalized to untreated controls.

Components of the NLRP3 inflammasome were assayed using Western Blotting as described^24^. THP-1 macrophages (3,000,000 cells seeded in a petri dish) were treated with MSU crystals (100 μg/mL) ± anti-CD44 or IC antibodies for 12 hours. Probing antibodies included anti-NLRP3 (rabbit monoclonal antibody), anti-caspase-1 (pro- and p10 subunit; rabbit monoclonal antibody), anti-ASC1 (rabbit polyclonal antibody) and anti-β actin (rabbit polyclonal antibody). All antibodies are commercially available (Abcam). Probing was performed using a 1:1,000 antibody dilution and blots were incubated with primary antibodies overnight. Following washing with TBS-T, secondary antibody (HRP-conjugated anti-rabbit goat polyclonal antibody; 1: 2,000 dilution; Abcam) was added for 1 hour at room temperature followed by washing with TBS-T. Blots were developed using a chemiluminescent substrate (ThermoFisher Scientific) followed by imaging using a Bio-Rad imager. Band intensities were determined using the Bio-Rad imager software. We used β-actin as our loading control and p10 casapase-1 and pro-caspase-1 band intensities were normalized to the band intensity of β-actin in the same sample, and expressed as a ratio normalized to untreated controls. Cellular pro-IL-1β and mature IL-1β levels were determined using ELISA kits (R&D Systems) and normalized to total protein level in the cell lysates.

Caspase-1 activity was determined following the incubation of THP-1 macrophages with MSU crystals ± anti-CD44 or IC antibodies for 12 hours as described above. Caspase-1 activity was determined using Caspase-Glo Assay Kit (Promega, USA) and expressed as relative luminescence units (RLUs).

### Intracellular protein phosphatase-2A (PP2A) activity in MSU-treated macrophages and the role of PP2A in mediating anti-CD44 antibody’s anti-inflammatory effect

THP-1 macrophages were treated with MSU crystals (100 μg/mL) ± anti-CD44 or IC antibodies, a PP2A activator (FTY720; Sigma) (2.5 μM), or a PP2A inhibitor (Okadaic acid; Sigma) (5 nM) for 2 hours followed by determination of intracellular PP2A activity (PP2A activity assay; Promega). To study the role of PP2A activity in mediating anti-CD44 antibody’s effect, THP-1 macrophages were treated with MSU crystals (100 μg/mL) ± anti-CD44, IC antibodies or anti-CD44 antibody + okadaic acid for 6 hours followed by determination of IL-1β gene expression and production as described above.

### Murine peritoneal model of acute gout

We employed the MSU peritoneal model of acute gout as previously described^20^. Male mice (12–14 weeks old) that were wildtype (n = 8), *Cd44*^*−/−*^ (n = 8) or *Nlrp3*^*−/−*^ (JAX stock # 021302) (n = 6)^41^ were injected via the intraperitoneal (IP) route with sterile PBS (200 μL) or MSU crystals (2 mg in 200 μL) under gas anesthesia. Prior to IP injections, abdomens were cleansed with 70% isopropyl alcohol. A total of 3 animals from each genotype were used as untreated controls. At 4 hours following injections, animals were euthanized using CO_2_ and animal abdomens were carefully opened without violating the integrity of the peritoneal cavity. Peritoneal lavaging was performed by injecting 4 mL of cold PBS into the peritoneal cavity followed by shaking for 30–60 seconds and lavage aspiration. Lavage fluids were centrifuged at 450 *g* for 10 min and supernatants were collected and assayed for IL-1β and interleukin-1 receptor antagonist (IL-1Ra) levels using ELISA kits (R&D Systems). Cell pellets were resuspended in 1 mL PBS and the total number of cells was determined using a hemocytometer.

Live cells were identified by side and forward scatter gating. For flow cytometry, cells were stained with a combination of anti-mouse Ly6b-FITC and Ly6G-APC to identify neutrophils^42^ or a combination of anti-mouse Cd11b-FITC and Ly6C-APC to identify monocytes^43^. Antibodies (Abcam) were used at 1:100 dilutions and incubated for 1 hour at 4 °C in the dark. Following staining, cells were washed with PBS and fixed in PBS containing 2% paraformaldehyde and analyzed using a flow cytometer. Infiltrating neutrophils were determined by multiplying the number of cells in the lavage by the percentage of cells that were Ly6b^+^/Ly6G^+^. Similarly, infiltrating monocytes were determined by multiplying the number of cells in the lavage by the percentage of cells that were Cd11b^+^/Ly6C^+^.

In another set of experiments, male wildtype mice (12–14 weeks old) were randomly assigned to the following experimental groups: untreated controls (n = 4), MSU + vehicle treated (n = 4), MSU + anti-CD44 antibody treated (n = 5) or MSU + IC antibody treated (n = 3). MSU crystals (2 mg in 200 μL), vehicle (PBS; 200 μL), anti-CD44 antibody (50 μg in 200 μL) or IC antibody (50 μg in 200 μL) injections were performed as described above. At 4 hours following injections, peritoneal lavaging and cell phenotypic analyses were conducted as described above. Peritoneal lavage IL-1β concentrations were determined using an ELISA kit (R&D Systems).

### Statistical analyses

Statistical analyses of gene expression data were performed using ΔCt values (C_t_ target gene-C_t_ GAPDH) for each gene of interest in each experimental group. We initially determined whether continuous variables satisfied the requirements for parametric statistical tests. Statistical significance comparing two groups or multiple groups with parametric data was assessed by Student’s *t* test or ANOVA followed by post-hoc multiple comparisons using Tukey’s post-hoc test. Statistical significance comparing two groups or multiple groups with nonparametric data was assessed by Rank Sum test or ANOVA on the ranks. A *p* value of <0.05 was considered statistically significant. Data are presented as scatter plots with mean and standard deviations highlighted. Data were generated from at least 3 independent experiments with duplicate wells per treatment.

## Results

### Phagocytosis of latex beads and MSU crystals, LDH release and downstream IL-1β expression and secretion were lower in *Cd44*^*−/−*^ BMDMs compared to *Cd44*^*+/+*^ BMDMs and a CD44 antibody treatment reduced MSU crystal uptake by murine macrophages

**Figure 1:**
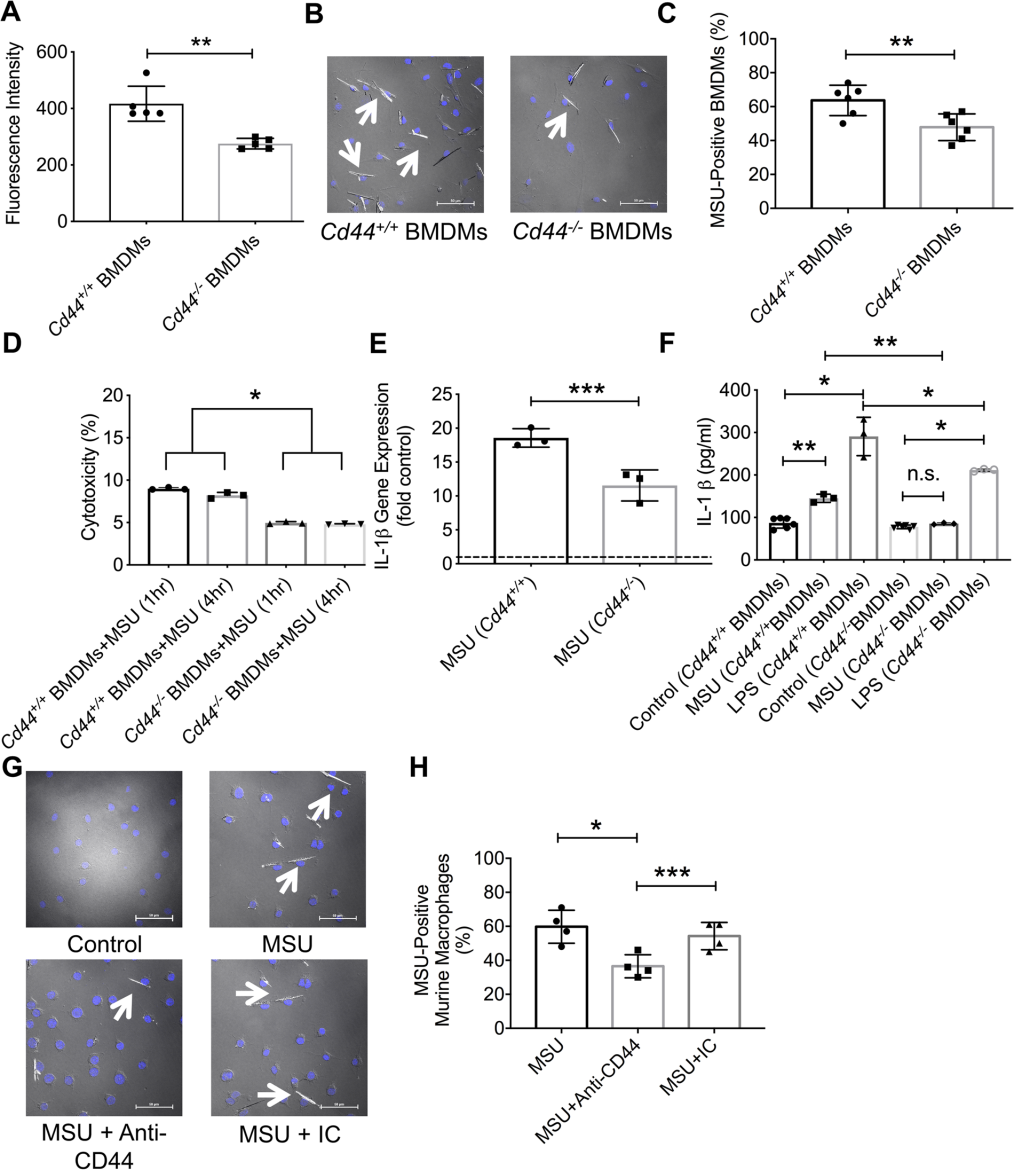
Role of CD44 receptor in mediating monosodium urate monohydrate (MSU) crystal phagocytosis and downstream inflammation in bone marrow derived macrophages (BMDMs) from *Cd44*^*+/+*^ and *Cd44*^*−/−*^ mice and impact of antibody-mediated receptor shedding on MSU phagocytosis by murine macrophages. BMDMs were treated with MSU (100 μg/mL) for 4 hours to evaluate phagocytosis and for 6 hours to evaluate interleukin-1 beta (IL-1β) gene expression and production. BMDMs were stimulated with lipopolysaccharide (LPS; 100 ng/ml) for 72 hours as a positive control. Lactate dehydrogenase (LDH) release from BMDMs was determined at 1 and 4 hours following incubation with MSU crystals and LDH activity level was used to calculate percent cytotoxicity. We utilized the IM7 clone, which recognizes all CD44 isoforms and causes shedding of the extracellular domain. Anti-CD44 and isotype control (IC) antibodies (2 μg/mL for both antibodies) were incubated with J774A.1 murine macrophages for 4 hours. Data from 3–5 independent experiments are presented with mean and S.D. highlighted. *p < 0.001; **p < 0.01; ***p < *0.05; n.s.: non significant*. Scale bar = 50 μm. Dashed line represents gene expression in untreated controls. (**a**) Phagocytosis of FITC-labeled antibody-coated beads was lower in *Cd44*^*−/−*^ BMDMs. (**b**) A representative image showing MSU crystal internalization in *Cd44*^*+/+*^ and *Cd44*^*−/−*^ BMDMs following incubation with MSU crystals for 4 hours. Arrows point to cells with internalized MSU crystals. (**c**) MSU phagocytosis was reduced in *Cd44*^*−/−*^ BMDMs. (**d**) Percent cytotoxicity was lower in *Cd44*^*−/−*^ BMDMs. (**e**) MSU-stimulated IL-1β gene expression was lower in *Cd44*^*−/−*^ BMDMs. (**f**) MSU and LPS-stimulated IL-1β secretion was lower in *Cd44*^*−/−*^ BMDMs. (**g**) Representative images depicting the impact of anti-CD44 antibody treatment on MSU phagocytosis by murine J774A.1 macrophages. Arrows point to cells with internalized MSU crystals. (**h**) Anti-CD44 antibody treatment reduced MSU phagocytosis by murine macrophages.

Absence of CD44 resulted in a significant reduction in the phagocytosis of latex beads and MSU crystals by murine BMDMs. As shown in Fig. 1A, *Cd44*^*−/−*^ BMDMs demonstrated a reduced uptake of latex beads compared to *Cd44*^*+/+*^ BMDMs (*p* < *0.01*). Similarly, *Cd44*^*−/−*^ BMDMs showed a significantly lower ability to internalize MSU crystals compared to *Cd44*^*+/+*^ BMDMs (Fig. 1B,C; *p* < *0.01*). To investigate whether the reduced MSU crystal uptake by *Cd44*^*−/−*^ BMDMs was due to a cytotoxic effect by the crystals, we studied LDH release by *Cd44*^*−/−*^ and *Cd44*^*+/+*^ BMDMs. *Cd44*^*−/−*^ BMDMs had a significantly lower percent cytotoxicity at both 1 and 4-hour MSU treatments compared to *Cd44*^*+/+*^ BMDMs (Fig. 1D; *p* < *0.001* for both comparisons). IL-1β expression in MSU-treated *Cd44*^*−/−*^ or *Cd44*^*+/+*^ BMDMs was higher than corresponding expression in untreated control BMDMs (Fig. 1E; *p* < *0.001*). In MSU-treated BMDMs, we observed a biologically and statistically significant attenuation of IL-1β expression in *Cd44*^*−/−*^ BMDMs relative to *Cd44*^*+/+*^ BMDMs that approximated 38% in magnitude (Fig. 1E; *p* < *0.05*). Furthermore, IL-1β secretion was lower in MSU-treated *Cd44*^*−/−*^ BMDMs compared to MSU-treated *Cd44*^*+/+*^ BMDMs (Fig. 1F; *p* < *0.01*). Treatment of *Cd44*^*−/−*^ BMDMs with LPS resulted in a lower mean IL-1β supernatant concentration compared to LPS-treated *Cd44*^*+/+*^ BMDMs (Fig. 1F; *p* < *0.001*). The contribution of CD44 to MSU phagocytosis by murine macrophages was further illustrated by a reduced MSU uptake in MSU + anti-CD44 antibody-treated macrophages compared to MSU alone (Fig. 1G,H; *p* < *0.001*) or MSU + IC antibody treatment (Fig. 1H; *p* < *0.05*). The antibody effect was specific to CD44 as the IC antibody treatment failed to alter MSU uptake by murine macrophages (Fig. 1H; *p* > *0.05*).

### Direct crystal visualization and indirect flow cytometry are valid methods to detect MSU crystal uptake by THP-1 macrophages and exposure to MSU crystals resulted in enhanced CD44 receptor expression and cytosolic CD44 localization

**Figure 2:**
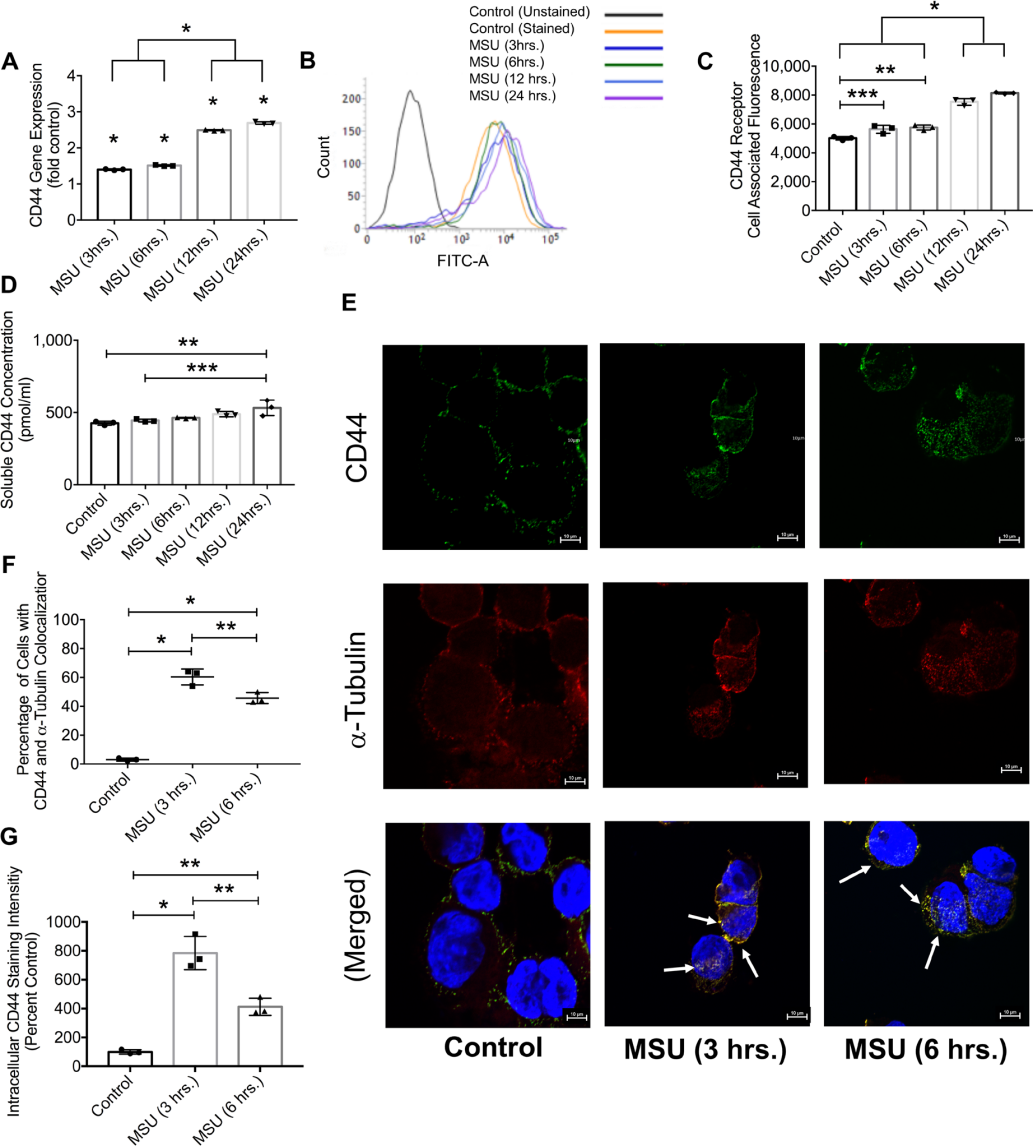
Impact of monosodium urate monohydrate (MSU) crystal treatment on CD44 receptor expression and internalization in differentiated human THP-1 macrophages. CD44 expression was determined using qPCR, flow cytometry and ELISA at 3, 6, 12 and 24 hours following MSU treatment. CD44 cytosolic internalization was determined using confocal microscopy, and intracellular CD44 staining intensity was determined following incubation with MSU crystals for 3 and 6 hours. Data are from 3 independent experiments and are presented with mean and S.D. highlighted. *p < 0.001; **p < 0.01; ***p < *0.05*. Scale bar = 10 μm. (**a**) CD44 expression exhibited a time-dependent increase following MSU crystal treatment. CD44 expression levels at 12 and 24 hours were higher than corresponding levels at 3 and 6 hours. CD44 expression in MSU-treated macrophages was higher than controls across all time points. (**b**) A representative flow cytometry histogram demonstrating elevated surface CD44 receptor protein on THP-1 macrophages following MSU crystal treatment. (**c**) Semi-quantitative analysis of mean CD44 receptor staining intensity following incubation with MSU crystals. Surface CD44 receptor protein levels were higher at 12 and 24 hours compared to corresponding values at 3 and 6 hours. CD44 protein surface staining increased following incubation with MSU across all time points. (**d**) Soluble CD44 receptor concentration increased following incubation with MSU crystals for 24 hours. (**e**) Representative images of CD44 receptor (green) and α-tubulin (red) colocalization in MSU-treated THP-1 macrophages at 3 and 6-hours following MSU treatment. In the merged panel, arrows point to CD44 receptor and α-tubulin colocalization indicating CD44 receptor internalization. Median colocalization images are presented. (**f**) CD44 and α-tubulin colocalized intracellularly at 3 and 6 hours following MSU treatment. (**g**) Intracellular CD44 staining intensity increased following MSU crystal treatment.

Crystals were detected intracellularly in MSU-treated THP-1 macrophages by direct visualization while MCF-7 cells showed no internalized crystals (Supplementary Fig. 1A). Using flow cytometry, we also detected MSU-positive THP-1 macrophages, while we failed to detect MSU-positive MCF-7 cells (Supplementary Fig. 1B,C). Over a 4-hour treatment period, MSU crystals were completely phagocytosed by THP-1 macrophages (Supplementary Video). As a result of MSU treatment, CD44 expression increased in a time-dependent manner (Fig. 2A; *p* < *0.001*). Over a 24-hour treatment period, cell surface CD44 protein staining also increased (Fig. 2B,C; *p* < *0.001*). In addition to surface receptor expression, mean soluble CD44 receptor concentration was higher after a 24-hour MSU treatment compared to untreated controls (Fig. 2D; *p* < *0.001*). Representative images showing CD44 receptor and α-tubulin (a cytosolic marker) staining following the incubation of THP-1 macrophages with MSU crystals for 3 or 6 hours are shown in Fig. 2E. Arrows point to the colocalization of CD44 receptor and α-tubulin. Macrophages demonstrated a positive intracellular colocalization of CD44 and α-tubulin at both time points with greater colocalization observed in the 3-hour group (Fig. 2F; *p* < *0.01* for all comparisons). There was little to no colocalization between CD44 and α-tubulin in control THP-1 macrophages, which was expected as CD44 is normally found on the cell surface. We also observed that the mean intracellular CD44 staining intensity at 3 or 6 hours was higher than corresponding mean value in control cells, with a higher mean intensity observed in the 3-hour group (Fig. 2G; *p* < *0.01* for all comparisons). We observed a 7.8-fold increase in intracellular CD44 staining intensity at 3 hours compared to control, wherein *de-novo* CD44 expression only increased by ~ 40% over the same treatment period. Therefore, the CD44 protein that we detected intracellularly at 3 hours was not newly synthesized but was likely internalized in response to MSU treatment.

### Antibody-mediated CD44 ECD shedding reduced MSU crystal phagocytosis, NF-κB p65 nuclear levels, conversion of procaspase-1 to caspase-1, and downstream IL-1β and IL8 expression mediated by intracellular PP2A activation

**Figure 3:**
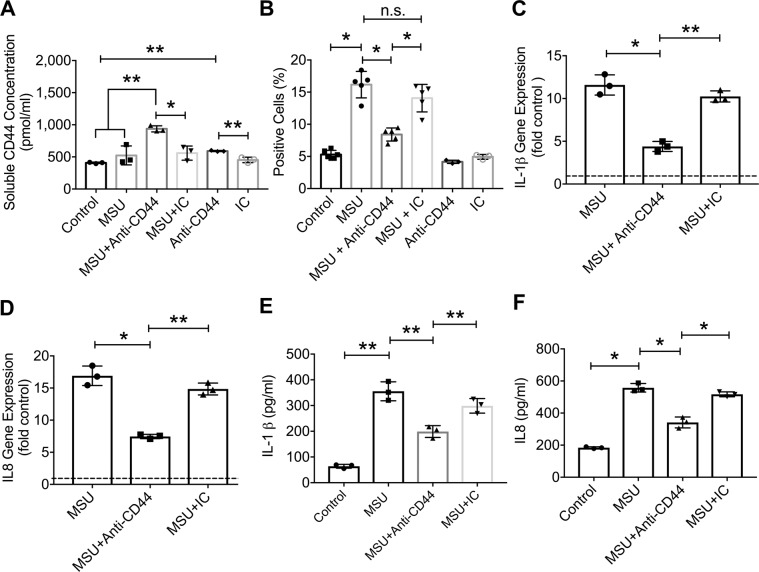
Impact of antibody-mediated CD44 receptor shedding on monosodium urate monohydrate (MSU) crystal phagocytosis by THP-1 macrophages, expression and production of interleukin-1 beta (IL-1β) and interleukin-8 (IL8). We utilized the IM7 clone, which recognizes all CD44 isoforms and causes enzyme-mediated shedding of the extracellular domain. THP-1 macrophages were incubated with MSU crystals (100 μg/mL) ± anti-CD44 or isotype control (IC) antibodies (2 μg/mL for both antibodies). MSU phagocytosis was determined by the change in side scatter in macrophages following a 4-hour incubation and expressed as percent positive cells that internalized MSU crystals. Representative flow cytometry plots are shown in supplementary figure 2. Gene expression and production of cytokines were determined following a 6-hour incubation. Data from 3–5 independent experiments are presented with mean and S.D. highlighted. Dashed line represents gene expression in untreated controls. *p < 0.001; **p < 0.01; ***p < *0.05; n.s.: non-significant*. (**a**) Anti-CD44 antibody treatment increased CD44 receptor shedding by macrophages. (**b**) Anti-CD44 antibody treatment reduced MSU phagocytosis by THP-1 macrophages. (**c**) Anti-CD44 antibody treatment reduced MSU-induced IL-1β expression. (**d**) Anti-CD44 antibody treatment reduced MSU-induced IL8 expression. (**e**) Anti-CD44 antibody treatment reduced MSU-induced IL-1β production. (**f**) Anti-CD44 antibody treatment reduced MSU-induced IL8 production.

We confirmed that the anti-CD44 antibody treatment induced CD44 ECD shedding in THP-1 macrophages (Fig. 3A; *p* < *0.01* between MSU + anti-CD44 and MSU alone). Antibody-mediated ECD shedding significantly reduced MSU phagocytosis by THP-1 macrophages (*p* < *0.001*) (Supplementary Fig. 2A through 2F and Fig. 3B). The lack of an anti-phagocytic effect by the IC antibody (Fig. 3B; *p* > *0.05* between MSU + IC and MSU alone) further confirms the role of CD44 in MSU crystal uptake by macrophages. Anti-CD44 antibody treatment reduced IL-1β expression (Fig. 3C; *p* < *0.001*) and production (Fig. 3E; *p* < *0.01*) in MSU-treated THP-1 macrophages with no effect by the IC antibody treatment (*p* > *0.05*). A similar effect was observed for IL-8 expression (Fig. 3D; *p* < *0.001*) and production (Fig. 3F; *p* < *0.001*).

**Figure 4:**
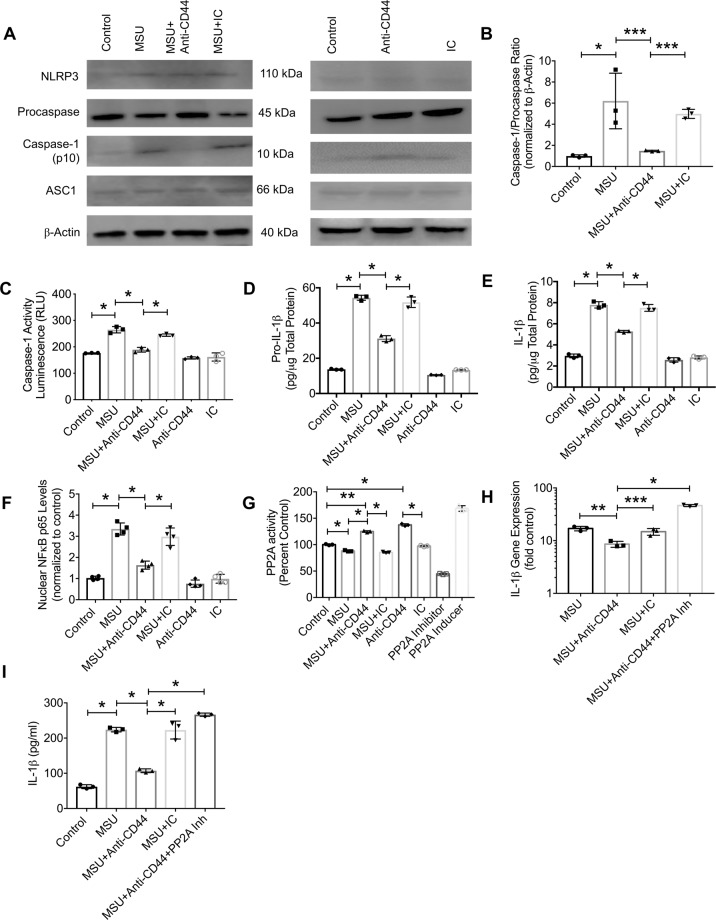
Impact of anti-CD44 antibody treatment on NLRP3 inflammasome activation and nuclear factor kappa B (NFκB) p65 subunit nuclear translocation in differentiated human THP-1 macrophages following incubation with monosodium urate monohydrate (MSU) crystals and role of intracellular protein-phosphatase 2A (PP2A) in mediating anti-CD44 antibody’s effect. We utilized the IM7 clone, which recognizes all CD44 isoforms and causes enzyme-mediated shedding of the extracellular domain. THP-1 macrophages were treated with MSU crystals (100 μg/mL) ± anti-CD44 or isotype control (IC) antibodies (2 μg/mL for both antibodies). NF-κB p65 nuclear levels were determined following a 1-hour incubation using a DNA binding assay. Pro-caspase-1 and the p10 subunit of active caspase-1 were determined using Western Blotting. Active caspase-1 activity was determined using a specific activity assay. Cellular pro-IL-1β and mature IL-1β levels were determined using an ELISA. PP2A inhibition was performed using okadiac acid (5 nM). Data from 3 independent experiments are presented with mean and S.D. highlighted. *p < 0.001; **p < 0.01; ***p < *0.05*. The representative Western Blots shown represent two separate experiments. One experiment included Control, MSU, MSU + Anti-CD44 antibody and MSU + IC antibody. The other experiment included Control, Anti-CD44 antibody and IC antibody. The different proteins that we probed for were run on separate gels using the same experimental samples. (**a**) Representative Western Blots of NLRP3 inflammasome components. Anti-CD44 antibody treatment reduced procaspase conversion to active caspase-1. (**b**) Anti-CD44 antibody treatment reduced MSU-induced generation of p10 subunit of active caspase-1 in macrophages. (**c**) Anti-CD44 antibody treatment reduced caspase-1 activity. (**d**) Anti-CD44 antibody treatment reduced intracellular pro-IL-1β levels. (**e**) Anti-CD44 antibody treatment reduced intracellular mature IL-1β levels. (**f**) Anti-CD44 antibody treatment reduced NF-κB p65 subunit nuclear levels. (**g**) Anti-CD44 antibody treatment increased intracellular PP2A activity. (**h**) Co-treatment with anti-CD44 antibody and a PP2A inhibitor increased IL-1β expression. (**i**) Co-treatment with anti-CD44 antibody and a PP2A inhibitor increased IL-1β production.

We investigated the impact of anti-CD44 antibody treatment on procaspase-1 conversion to active caspase-1, caspase-1 activity and assayed pro-IL-1β and mature IL-1β levels in THP-1 cell lysates. Anti-CD44 antibody treatment reduced the conversion of procasapase-1 to active caspase-1 as demonstrated by a lower p10 caspase-1 subunit/procaspase-1 ratio in MSU + anti-CD44 antibody group compared to MSU alone (Fig. 4A,B; *p* < *0.01*). In addition, anti-CD44 antibody treatment reduced caspase-1 activity in MSU-challenged macrophages (Fig. 4C; *p* < *0.001* between MSU + anti-CD44 antibody and MSU alone). A consequence of reducing caspase-1 activity is a reduction in the generation of mature IL-1β. In this regard, anti-CD44 antibody treatment reduced cellular pro-IL-1β (Fig. 4D; *p* < *0.001*) and mature IL-1β (Fig. 4E; *p* < *0.001*) levels. Anti-CD44 antibody treatment also reduced NF-κB p65 nuclear levels in MSU-challenged THP-1 macrophages (Fig. 4F; *p* < *0.001* between MSU + anti-CD44 antibody and MSU alone). MSU treatment reduced intracellular PP2A activity in THP-1 macrophages compared to untreated controls (Fig. 4G; *p* < *0.001*). The addition of the anti-CD44 antibody increased intracellular PP2A activity in MSU-treated and untreated macrophages with no observed effect by the IC antibody (*p* < *0.001*). The magnitude of PP2A activity enhancement by anti-CD44 antibody was comparable to the positive control treatment. PP2A activation contributed to the anti-inflammatory effect seen with anti-CD44 antibody treatment. This is illustrated by significantly higher IL-1β expression (Fig. 4H; *p* < *0.001*) and production (Fig. 4I; *p* < *0.001*) in MSU + anti-CD44 antibody + PP2A inhibitor treatment compared to MSU + anti-CD44 antibody treatment.

### Intraperitoneal administration of MSU crystals did not trigger an inflammatory response in *Cd44*^*−/−*^ mice and anti-CD44 antibody treatment reduced neutrophil and monocyte recruitment and lavage IL-1β levels in a peritoneal model of gout

**Figure 5:**
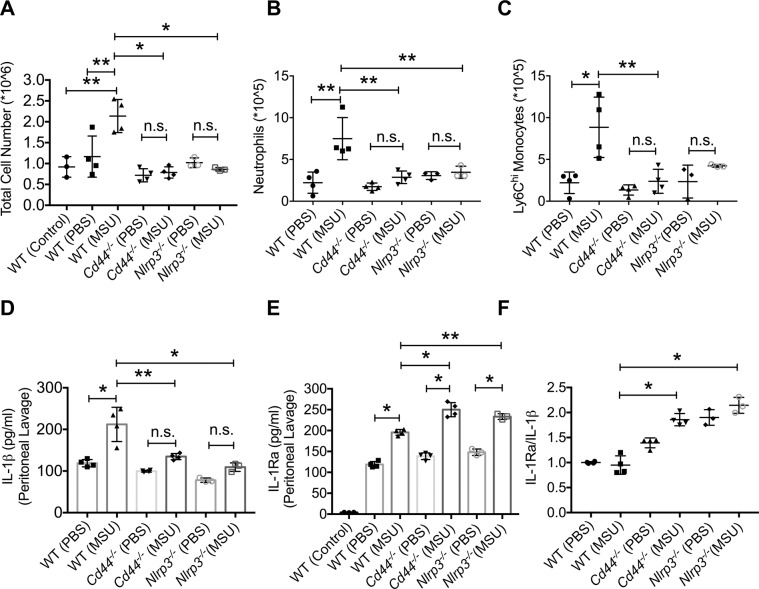
Impact of intraperitoneal administration of monosodium urate monohydrate (MSU) crystals on inflammatory cell infiltration and production of interleukin-1 beta (IL-1β) and interleukin-1 receptor antagonist (IL-1Ra) in wildtype, *Cd44*^*−/−*^ and *Nlrp3*^*−/−*^ mice. MSU (2 mg in 200 μl PBS) or PBS (200 μl) were administered via the intraperitoneal route in wildtype (n = 8), *Cd44*^*−/−*^ (n = 8) or *Nlrp3*^*−/−*^ (n = 6) and peritoneal lavaging was performed at 4 hours. Total peritoneal lavage cell counts were determined. The number of infiltrated neutrophils and monocytes were determined using flow cytometry and probing for neutrophil markers; Ly6B.2 and Ly6G and monocyte markers; Cd11b and Ly6C. Representative flow cytometry plots of neutrophil markers are shown in supplementary figure 3. Peritoneal lavage IL-1β and IL-1Ra levels were determined by ELISA and the IL-1Ra/IL-1β ratios were computed. *p < 0.001; **p < 0.01; ***p < *0.05*. (**a**) MSU administration in *Cd44*^*−/−*^ mice did not result in an increase in overall inflammatory cell infiltration. (**b**) MSU administration in *Cd44*^*−/−*^ mice did not result in increased neutrophil infiltration. (**c**) MSU administration in *Cd44*^*−/−*^ mice did not result in increased Ly6C^hi^ monocyte infiltration. (**d**) MSU administration in *Cd44*^*−/−*^ mice did not result in increased peritoneal IL-1β levels. (**e**) MSU administration in *Cd44*^*−/−*^ mice increased peritoneal IL-1Ra production. (**f**) Lavage IL-1Ra/IL-1β ratio was higher in *Cd44*^*−/−*^ mice.

Intraperitoneal administration of MSU crystals resulted in a significant increase in the number of cells recovered in lavages from wildtype mice compared to PBS-administered or untreated control counterparts (Fig. 5A; *p* < *0.01* for both comparisons). In contrast, administration of MSU crystals did not change the number of cells recovered in lavages of *Cd44*^*−/−*^ or *Nlrp3*^*−/−*^ mice (*p* > *0.05*). Furthermore, cell numbers in peritoneal lavages of MSU-administered wildtype mice were higher than corresponding numbers in *Cd44*^*−/−*^ and *Nlrp3*^*−/−*^ mice (*p* < *0.001* for both comparisons). Representative flow cytometry plots of neutrophils in peritoneal lavages are shown in Supplementary Fig. 3. Infiltrated neutrophils were higher in numbers in lavages of MSU-administered wildtype mice compared to PBS-administered animals (Fig. 5B; *p* < *0.01*). In contrast, infiltrated neutrophils did not increase in lavages of *Cd44*^*−/−*^ or *Nlrp3*^*−/−*^ mice following MSU administration (*p* > *0.05* for both comparisons). The number of infiltrated neutrophils was higher in lavages of MSU-administered wildtype mice compared to lavages of MSU-administered *Cd44*^*−/−*^ and *Nlrp3*^*−/−*^ mice (*p* < *0.01* for both comparisons). A similar genotype-dependent trend was observed for inflammatory monocytes in peritoneal lavages following intraperitoneal MSU administration (Fig. 5C). These findings indicate that both *Cd44*^*−/−*^ and *Nlrp3*^*−/−*^ genotypes showed a blunted inflammatory response towards MSU crystals, highlighted by the lack of neutrophil and monocyte recruitment to the peritoneum.

Lavage IL-1β levels in MSU-administered wildtype mice were higher than corresponding levels in PBS-administered mice (Fig. 5D; *p* < *0.001*). Lavage IL-1β levels were also higher in MSU-administered wildtype mice compared to MSU-administered *Cd44*^*−/−*^ (*p* < *0.01*) or *Nlrp3*^*−/−*^ (*p* < *0.001*) mice. Intraperitoneal administration of MSU increased lavage IL-1Ra levels in wildtype, *Cd44*^*−/−*^ and *Nlrp3*^*−/−*^ mice compared to PBS (Fig. 5E; *p* < *0.001* for all comparisons). Lavage IL-1Ra levels in MSU-administered wildtype mice were lower than corresponding levels in *Cd44*^*−/−*^ mice (*p* < *0.001*) or *Nlrp3*^*−/−*^ mice (*p* < *0.01*). The mean lavage IL-1Ra/IL-1β ratio in MSU-administered *Nlrp3*^*−/−*^ and *Cd44*^*−/−*^ mice was higher than the corresponding ratio in MSU-administered wildtype mice (Fig. 5F; *p* < *0.001* for both comparisons). IL-1Ra is the endogenous antagonist to IL-1β, and functions to regulate the inflammatory response. The higher IL-1Ra levels in MSU-administered *Cd44*^*−/−*^ and *Nlrp3*^*−/−*^ mice would therefore contribute to the lack of inflammatory response in both genotypes.

**Figure 6:**
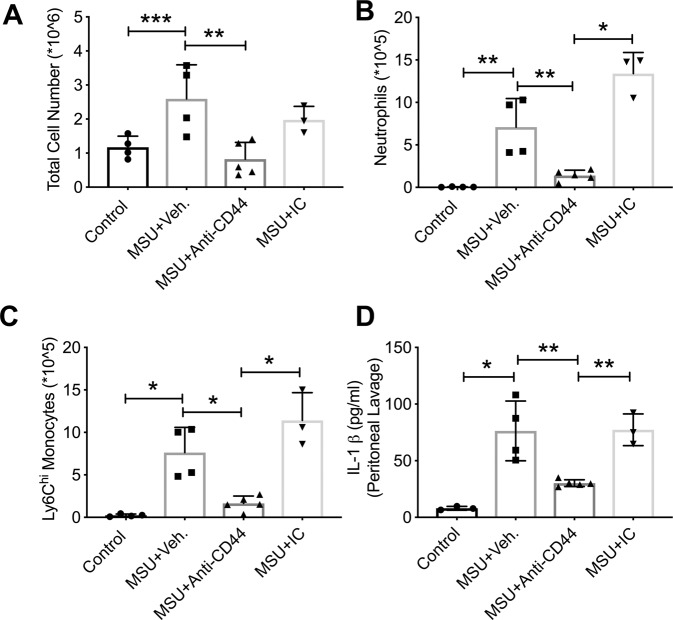
Impact of anti-CD44 antibody treatment on inflammatory cell infiltration and production of interleukin-1 beta (IL-1β) in murine peritoneal monosodium urate monohydrate (MSU) crystal inflammation model. MSU crystals (2 mg in 200 μL PBS), vehicle (Veh.; 200 μL PBS), anti-CD44 and isotype control (IC) antibodies (50 μg in 200 μL PBS) were administered via the intraperitoneal route. Experimental groups included untreated controls (n = 4), MSU + Veh. (n = 4), MSU + Anti-CD44 antibody (n = 5) and MSU + IC antibody (n = 3). Peritoneal lavaging was performed at 4 hours. Total peritoneal lavage cell counts were determined. The number of infiltrated neutrophils and monocytes were determined using flow cytometry and probing for neutrophil markers; Ly6B.2 and Ly6G and monocyte markers; Cd11b and Ly6C. Representative flow cytometry plots of neutrophil and monocytes markers are shown in Supplementary Fig. 4. Peritoneal lavage IL-1β levels were determined by ELISA. *p < 0.001; **p < 0.01; ***p < *0.05*. (**a**) Anti-CD44 antibody treatment reduced the number of cells in peritoneal lavages following MSU administration. (**b**) Anti-CD44 antibody treatment reduced neutrophil infiltration in MSU peritoneal inflammation model. (**c**) Anti-CD44 antibody treatment reduced Ly6C^hi^ monocyte infiltration in MSU peritoneal inflammation model. (**d**) Anti-CD44 antibody treatment reduced lavage IL-1β levels compared to vehicle or IC antibody treatments.

Anti-CD44 antibody treatment reduced the number of cells in peritoneal lavages of MSU-administered wildtype mice compared to vehicle treatment (Fig. 6A; *p* < *0.01*). Representative flow cytometry plots depicting neutrophils and monocytes in lavages from the different experimental groups are shown in Supplementary Fig. 4. Anti-CD44 antibody treatment reduced the number of neutrophils (Fig. 6B; *p* < *0.01*; *p* < *0.001*) and inflammatory monocytes (Fig. 6C; *p* < *0.001* for both comparisons) in peritoneal lavages of MSU-administered wildtype mice compared to vehicle or IC antibody treatments. Lavage IL-1 β levels in MSU + anti-CD44 antibody treatment were lower than corresponding levels in MSU + vehicle or MSU + IC antibody treatments (Fig. 6D; *p* < *0.01* for both comparisons).

## Discussion

In this study, we investigated the role of the CD44 receptor in mediating urate crystal phagocytosis by macrophages of different origins and examined the associated signaling pathway triggered by shedding the receptor’s extracellular domain. Urate crystals were uptaken by CD44-competent macrophages to a greater extent than what was observed in macrophages that lacked CD44. The reduced crystal phagocytosis by CD44 deficient macrophages resulted in reduced cell toxicity. Furthermore, blunted crystal phagocytosis was associated with attenuated induction of IL-1β expression and production by CD44 deficient macrophages. Interestingly, the magnitude of reduction in crystal phagocytic uptake by murine macrophages due to CD44 deficiency paralleled the magnitude of reduction in IL-1β expression and was comparable to the inflammatory response of TLR2 and TLR4 deficient macrophages^22^. The involvement of CD44 in the uptake of urate crystals by murine macrophages was further confirmed by the observed reduction in crystal internalization as a result of antibody-mediated receptor shedding. The observation that CD44 is a phagocytic receptor^44^ is further confirmed by our finding that CD44 deficient macrophages had a lower phagocytic activity against latex beads compared to CD44 competent macrophages. In human macrophages, CD44 translocated to the cytosol in response to urate crystals. Receptor translocation occurred rapidly, coincided with crystal uptake and was detectable up to 6 hours following urate crystal exposure. Antibody-mediated CD44 extracellular domain shedding resulted in a significant reduction in crystal uptake by human macrophages and downstream reduction in proinflammatory cytokine and chemokine expression and production. The reduction in IL8 expression and production is particularly relevant due to the important role of the IL8/CXCR-2 axis in mediating neutrophil infiltration in models of gout^45–47^. The observed effect of the antibody was specific to CD44 as the isotype control antibody lacked any appreciable anti-phagocytic or anti-inflammatory activities. Our results suggest that CD44’s extracellular domain recognizes and binds naked urate crystals resulting in the internalization of the crystal-receptor complex. The cleavage of the extracellular domain thus reduced the number of available binding sites for the urate crystals, which translated into lower crystal phagocytosis. While urate crystals were reported to non-specifically bind more than 20 plasma proteins^22,48,49^, their interaction with CD44 appears to be biologically significant in the context of how macrophages respond to urate crystal danger signals.

IL-1β is a potent multifunctional proinflammatory cytokine that is pivotal in acute gout and clinically available IL-1 inhibitors, e.g. recombinant human IL-1Ra, were shown to be effective in relieving signs and symptoms of acute gout flares^50^. Unlike other proinflammatory cytokines *e.g*. TNF-α and IL-6, IL-1β is initially produced as a biologically inactive precursor, pro-IL-1β as a result of priming of monocytes and/or macrophages^21^. Pro-IL-1β requires further intracellular processing by caspase-1 for the generation of mature IL-1β^21^. Priming of macrophages can occur as a result of exposure to TLR ligands including MSU crystals^21^. In our study, exposure to MSU crystals resulted in *priming* and *activation* of murine and human macrophages. MSU crystals enhanced the nuclear translocation of NF-κB p65 subunit, increased pro-IL-1β production and enhanced the conversion of procaspase to activated caspase-1 resulting in increased intracellular caspase-1 activity and secreted mature IL-1β levels. CD44 appears to play a role in regulating macrophage priming by TLR2 and TLR4 agonists as evidenced by our prior^34^ and current finding that CD44-deficient macrophages were not as responsive towards Pam3CSK4 (A TLR2 ligand) or LPS (a TLR4 ligand) stimulation as CD44 competent macrophages. Antibody-mediated CD44 extracellular domain shedding blocked the priming signal by MSU crystals mediated by reduced NF-κB activation, resulting in reduced IL-1β gene expression and pro-IL-1β production. Related to NLRP3 inflammasome, anti-CD44 antibody reduced procaspase conversion to caspase-1 which reduced mature IL-1β generation. The inhibitory effect of anti-CD44 antibody on NLRP3 inflammasome activity was a downstream effector of its blocking of MSU uptake by macrophages. These effects were specific for targeting the CD44 receptor as the isotype control antibody showed no effect on NLRP3 inflammasome activation.

The role of CD44 in regulating macrophage priming by urate crystals is independent of its role in facilitating their phagocytosis. Urate crystals enhanced CD44 gene expression and production, consistent with the upregulation of CD44 expression in the setting of inflammation and in response to innate immune signals^34^. CD44 is coupled to multiple signaling pathways including non-receptor *Src* tyrosine kinases, activators of small Rho GTPases and PP2A^34,51–53^. The type of signaling pathway coupled to CD44 is cell specific and depends on the ligand that engages the receptor^54^. In addition, the cytoplasmic tail of the receptor can be cleaved off by γ-secretase where it translocates to the nucleus and acts as a transcriptional regulator^54^. PP2A is a member of the intracellular serine/threonine phosphatase family that plays a counter-regulatory role in deactivating phosphorylation-dependent pathways involved in innate immune macrophage activation^55,56^. Gene deletion of PP2A was shown to magnify the inflammatory response of peritoneal macrophages due to LPS^56^ and we have reported that an anti-CD44 antibody reduced TLR2 ligand induced inflammation in bone marrow derived macrophages mediated by intracellular PP2A activation^34^. Consistent with their role as an innate immune danger signal, urate crystals reduced intracellular PP2A activity while anti-CD44 antibody enhanced cytosolic PP2A activity in cells that were treated with the antibody alone or a combination of the crystals and the antibody. The increase in PP2A activity as a result of CD44 extracellular domain shedding was similar to the enhancement in PP2A activity by the PP2A-activating drug FTY720^57^. The anti-inflammatory activity of anti-CD44 antibody was mediated by enhanced PP2A activity as co-treatment with a PP2A inhibitor reversed the effect of the antibody and exacerbated the inflammatory response of macrophages towards urate crystals.

Neutrophil and monocyte transendothelial recruitment and activation are critical to the onset of inflammation in acute gout^5,21^. Crystal-induced neutrophil influx was shown to be CXCR2-dependent and relied on NLRP3 inflammasome activation *in-vivo*^58^. The interaction between neutrophils and urate crystals results in the phagocytosis of these crystals by neutrophils and the secretion of proinflammatory cytokines and chemokines that recruit more neutrophils to the site of inflammation^59,60^. In addition, apoptosis of neutrophils by the phagocytosed crystals results in formation of neutrophil extracellular traps (NETs), which continue to release proinflammatory cytokines^60^. As inflammation progresses and in the presence of high density of neutrophils, aggregation of NETs takes place and these newly formed aggregated NETs exert an anti-inflammatory effect due to their ability to degrade proinflammatory cytokines *e.g*. IL-1β and IL-6^59,60^. In our peritoneal gout model, we demonstrated enhanced recruitment of neutrophils and monocytes following a urate crystal challenge, similar to what was previously reported^20,22^. Recruitment of neutrophils and monocytes was associated with increased IL-1β secretion in the peritoneum. In contrast to CD44-competent animals, CD44 deficient mice exhibited a blunted neutrophil and monocyte recruitment with approximately 60% reduction in number of infiltrated neutrophils and monocytes. Common between CD44 and NLRP3-deficient mice are the lack of inflammatory cell infiltrates, blunted generation of IL-1β and higher IL-Ra levels. IL-1Ra is an important negative modulator of inflammation and the higher IL-1Ra levels in CD44-deficient mice contributes to the resistance of this phenotype towards crystal-induced inflammation. The mechanism by which the lack of CD44 drives higher IL-1Ra expression remains unclear. IL-1Ra and IL-1β were shown to be regulated in opposite directions by the phosphatidylinositide-3 kinase (PI3K) pathway in monocytes and macrophages^61^. Furthermore, peroxisome-proliferator-activated receptor gamma (PPARγ) ligands upregulated IL-1Ra expression in THP-1 macrophages^62^. Since CD44 regulates different signaling pathways including PI3K/AKT pathway^30^, the enhanced expression of IL-1Ra in CD44-deficient macrophages might be related to increased activation of the PI3K pathway in the absence of CD44. Targeting CD44 in wildtype animals had a biologically significant anti-inflammatory effect and recapitulated the CD44-deficient phenotype, evidenced by reduced neutrophils and monocyte recruitment and attenuated IL-1β generation. Taken together, our *in-vivo* findings strongly support that CD44 is essential to the pathogenesis of gout, and pharmacological manipulation of its role is efficacious in models of acute urate crystal induced inflammation.

In conclusion, we investigated the role of CD44 in mediating urate crystal induced inflammation *in-vitro* and *in-vivo*. CD44 mediated urate crystal phagocytosis with intracellular translocation of the receptor as a result of exposure to these crystals. Antibody-mediated CD44 extracellular domain shedding reduced urate crystal phagocytosis in murine and human macrophages. CD44 extracellular domain shedding also reduced urate crystal induced NF-κB nuclear translocation and pro-IL-1β production. Conversion of procaspase to caspase-1, caspase-1 activity and secretion of mature IL-1β were also inhibited by antibody-mediated CD44 extracellular domain shedding. CD44 deficiency protected mice from inflammation and recruitment of neutrophils and monocytes in a peritoneal model of acute gout. Anti-CD44 antibody treatment abrogated the recruitment of neutrophils and monocytes and reduced inflammation in the same model. The CD44 receptor plays a significant role in the pathogenesis of urate crystal inflammation and pharmacological strategies that target this receptor present a novel approach to treat acute gout.

## Supplementary information

Supplementary Figures.

Supplementary Video.
