# Supplementary material for: CD44 Receptor Mediates Urate Crystal Phagocytosis by Macrophages and Regulates Inflammation in A Murine Peritoneal Model of Acute Gout
Source: Sci Rep. 2020 Apr 1;10:5748. doi: 10.1038/s41598-020-62727-z (PMC7113258; doi:10.1038/s41598-020-62727-z)
Supplement: Supplementary file 1 — Supplementary Figures. [file 41598_2020_62727_MOESM1_ESM.pdf]

CD44 Receptor Mediates Urate Crystal Phagocytosis by Macrophages and Regulates Inflammation in A Murine Peritoneal Model of Acute Gout

[1,2]Emira Bousoik, [1,3]Marwa Qadri and [1]Khaled A. Elsaid

[1]Department of Biomedical and Pharmaceutical Sciences, Chapman University School of Pharmacy, Chapman University, CA, USA
[2]School of Pharmacy, Omar-Al-Mukhtar University, Derna, Libya
[3]Department of Pharmacology, College of Pharmacy, Jazan University, Jazan 82826, Saudi Arabia

Supplementary Figures

Supplementary Figure 1 Comparative phagocytic activities of human THP-1 macrophage and breast cancer cell line (MCF-7) against monosodium urate monohydrate (MSU) crystals using direct visualization and indirect flow cytometry side-scatter. Using flow cytometry, MSU phagocytosis was determined by the change in side scatter distribution of cells (THP-1 or MCF-7) following a 4-hour incubation. Two regions of interest were identified; P1 representing the cell population in the absence of MSU exposure and P2 representing the cell population with increased side scatter due to MSU phagocytosis. The percentage of positive cells was calculated as the number of cells in P2 region divided by the number of cells in P1+P2 regions. *$p<0.001$*; *n.s.*: non-significant; Scale bar = 25μm. **A)** Representative images showing crystals in MSU-treated THP-1 macrophages (white arrows) while no crystals were detected in MSU-treated MCF-7 cells. **B)** Representative flow cytometry plots of control THP-1 cells, MSU-treated THP-1 cells, control MCF-7 cells and MSU-treated MCF-7 cells showing localization of THP-1 cells in P2 region due to MSU phagocytosis. **C)** MSU phagocytosis was observed in THP-1 macrophages using side-scatter analysis but not in MCF-7 cells.

A

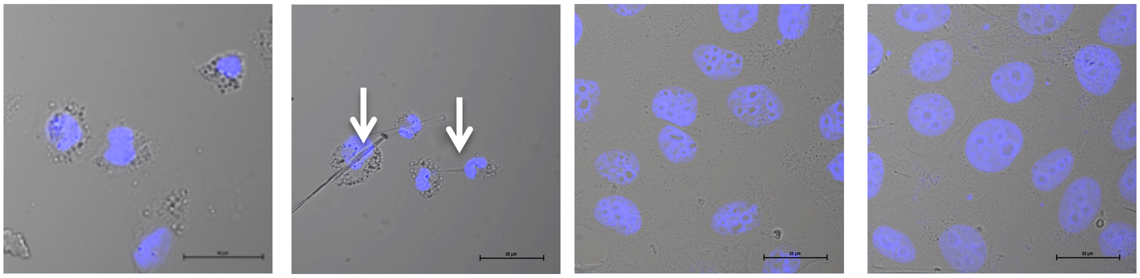

Control (THP-1) | MSU (THP-1) | Control (MCF-7) | MSU (MCF-7)

B

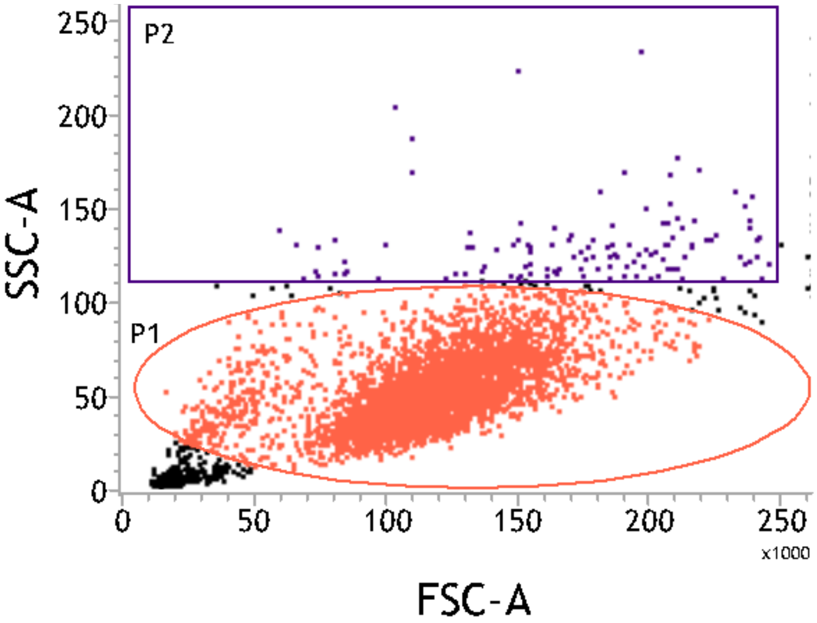

Control (THP-1)

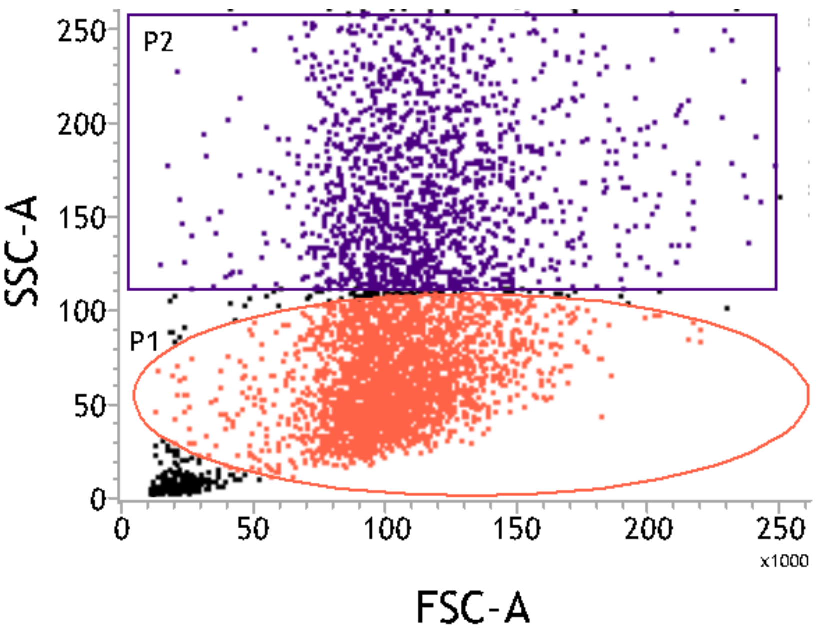

MSU (THP-1)

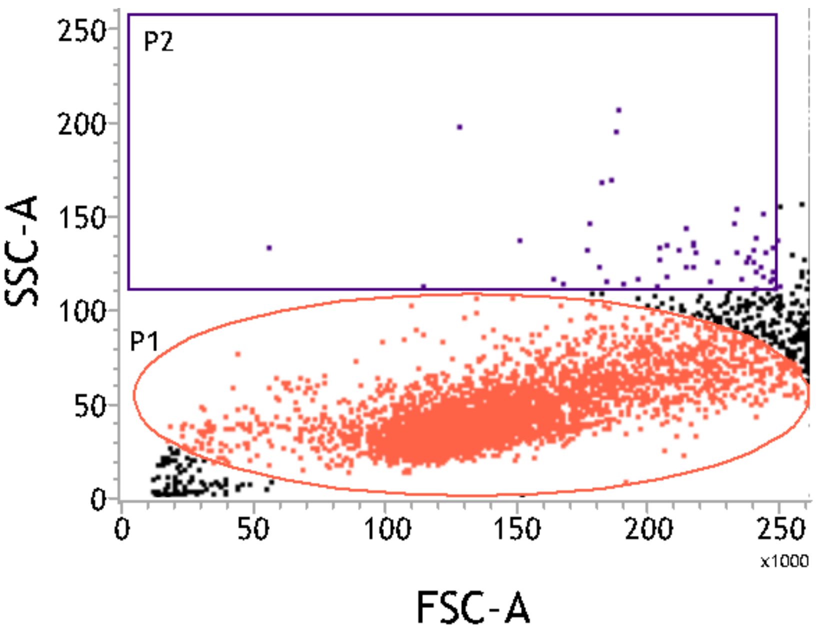

Control (MCF-7)

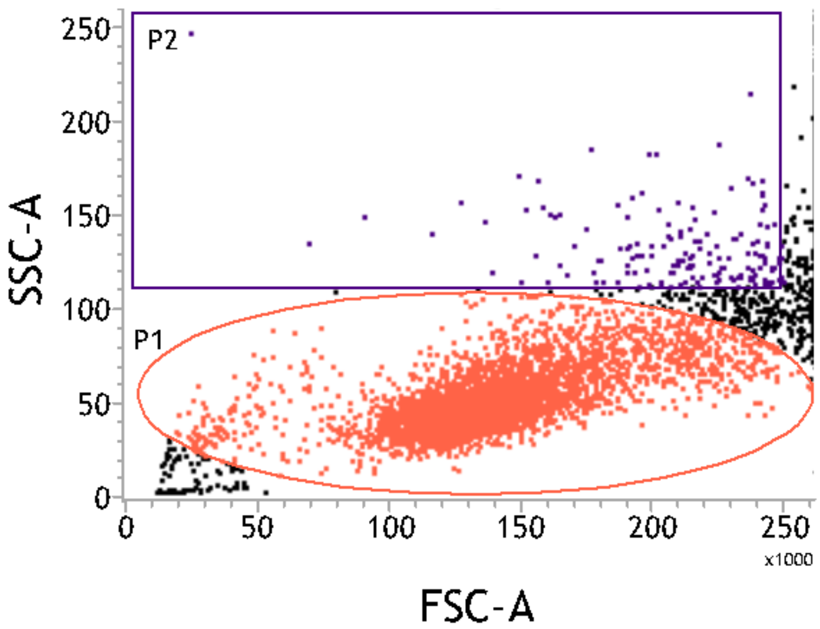
P2
P1
SSC-A
FSC-A
x1000
0
50
100
150
200
250

MSU (MCF-7)

C

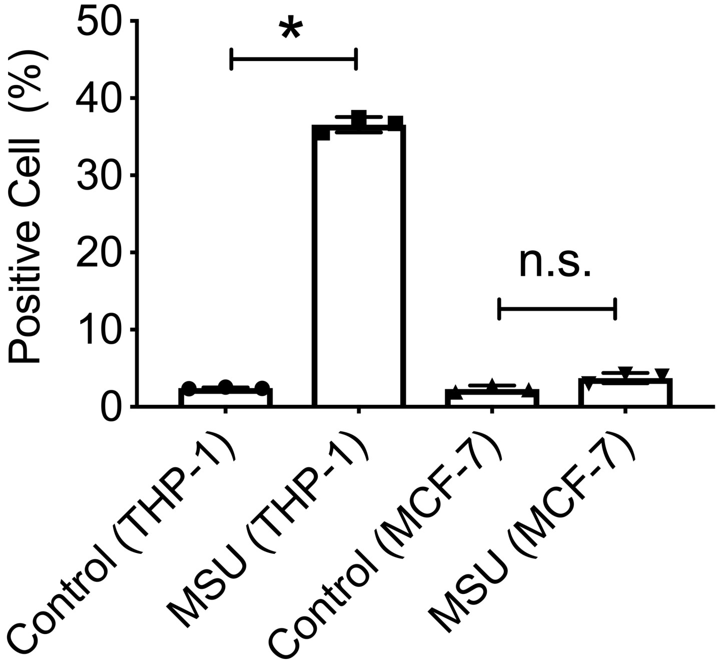
Positive Cell (%)
*
n.s.
Control (THP-1)
MSU (THP-1)
Control (MCF-7)
MSU (MCF-7)
0
10
20
30
40
50

Supplementary Figure 2 Representative flow cytometry plots depicting the impact of antibody-mediated CD44 receptor shedding on monosodium urate monohydrate (MSU) crystals phagocytosis by differentiated human THP-1 macrophages. MSU phagocytosis was determined by the change in side scatter distribution of macrophages following a 4-hour incubation. Two regions of interest were identified; P1 representing the macrophage population in the absence of MSU exposure and P2 representing the macrophage population with increased side scatter due to MSU phagocytosis in the presence or absence of anti-CD44 or isotype control (IC) antibodies (2µg/mL for both antibodies). **A)** Control; **B)** MSU; **C)** MSU+Anti-CD44; **D)** MSU+IC; **E)** Anti-CD44 and **F)** IC.

A

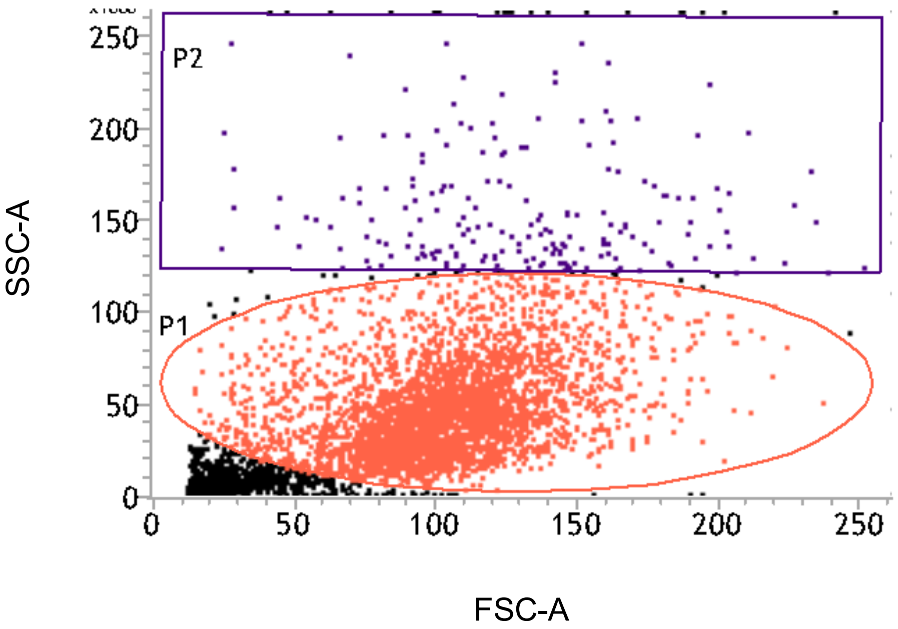

Control

B

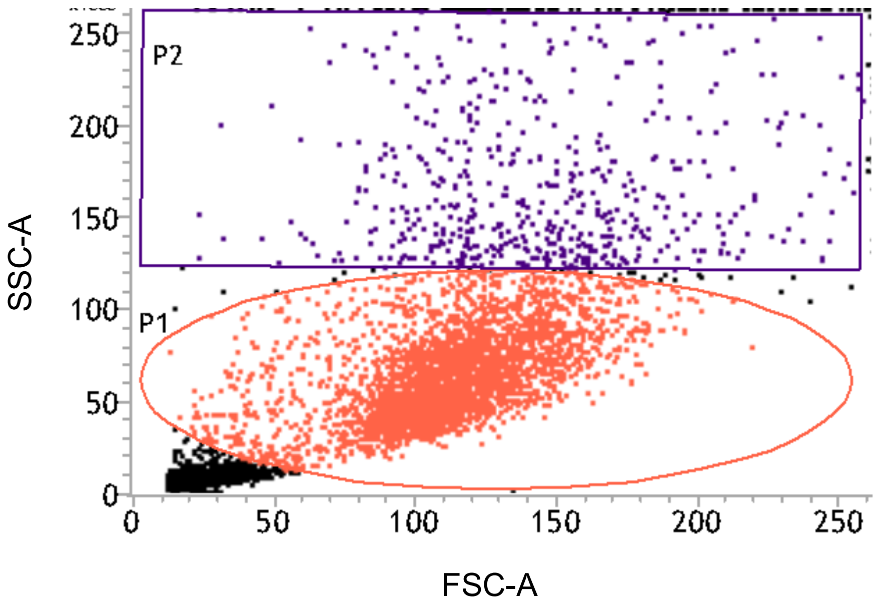

MSU

C

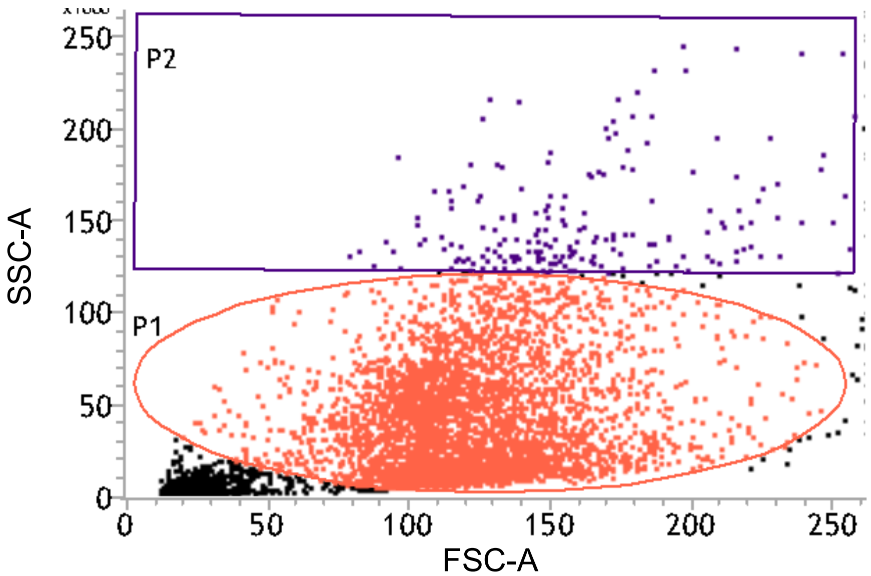

MSU+Anti-CD44

D

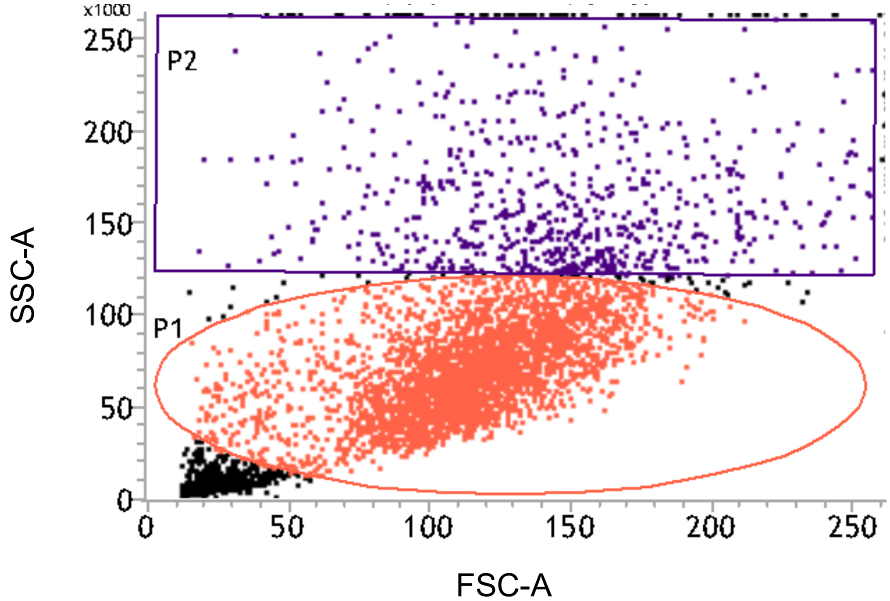

MSU+IC

E

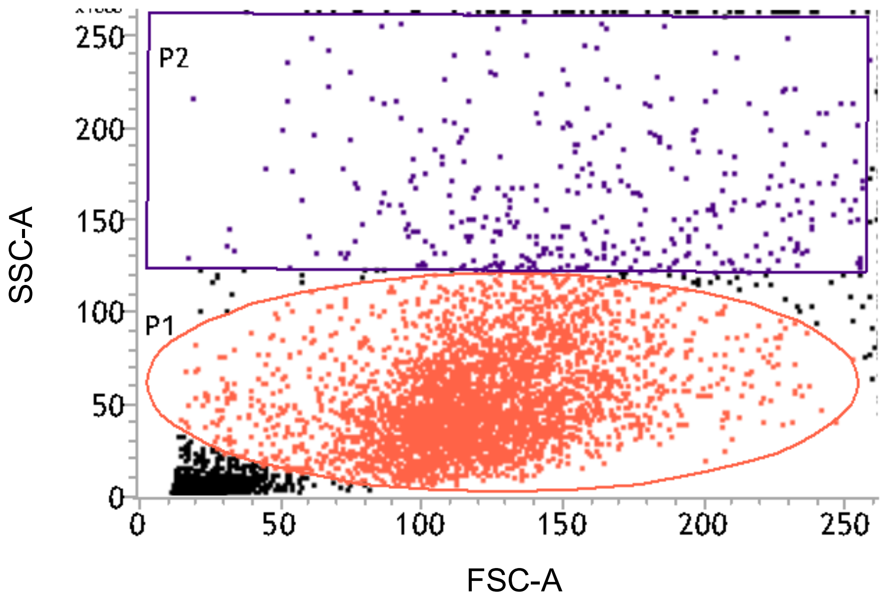

Anti-CD44

F

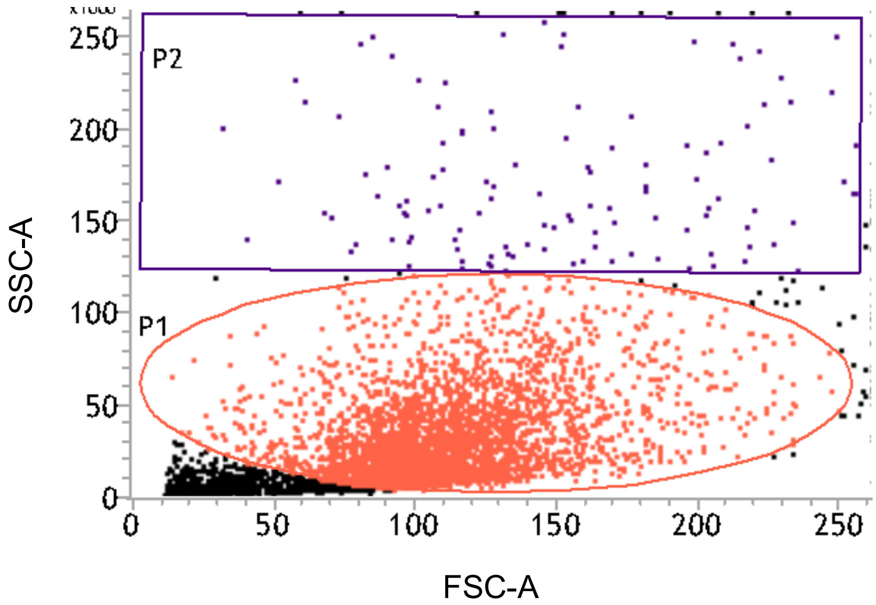

IC

Supplementary Figure 3 Representative flow cytometry plots showing increased percentage of lavage cells positive for Ly6B.2 and Ly6G in MSU-administered wildtype (WT) mice compared to *Cd44*$^{-/-}$ and *Nlrp3*$^{-/-}$ mice. **A)** Control, WT; **B)** Control, *Cd44*$^{-/-}$; **C)** Control, *Nlrp3*$^{-/-}$; **D)** PBS, WT; **E)** PBS, *Cd44*$^{-/-}$; **F)** PBS, *Nlrp3*$^{-/-}$; **G)** MSU, WT; **H)** MSU, *Cd44*$^{-/-}$; **I)** MSU, *Nlrp3*$^{-/-}$.

A

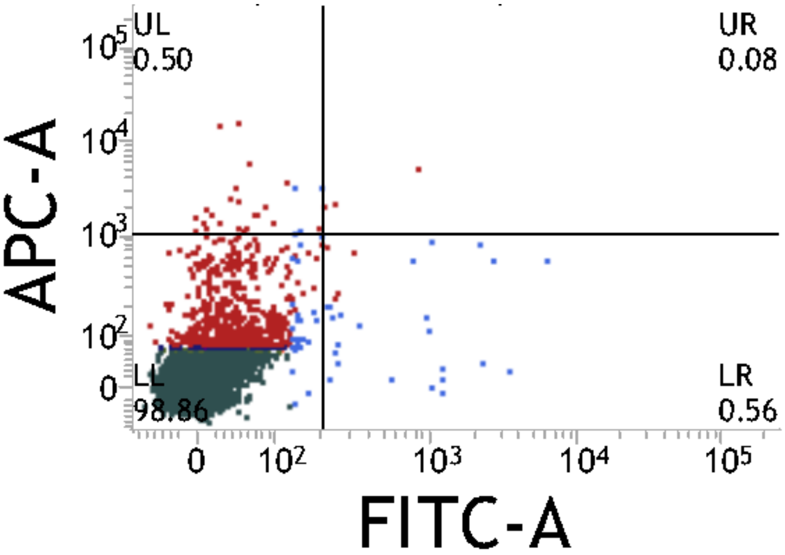

B

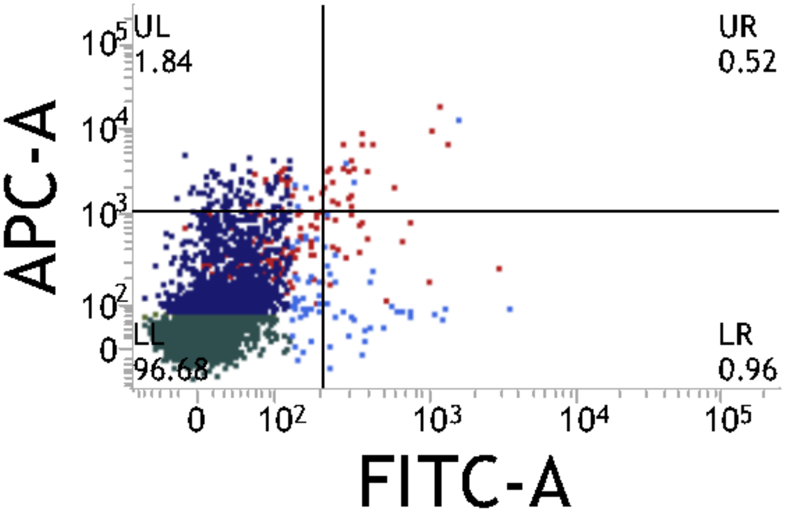

C

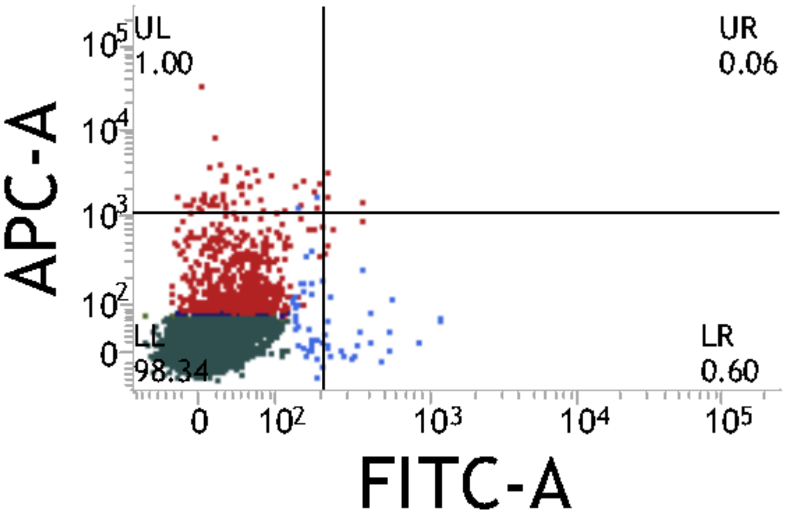
UL
1.00
UR
0.06
LL
98.34
LR
0.60
APC-A
FITC-A

D

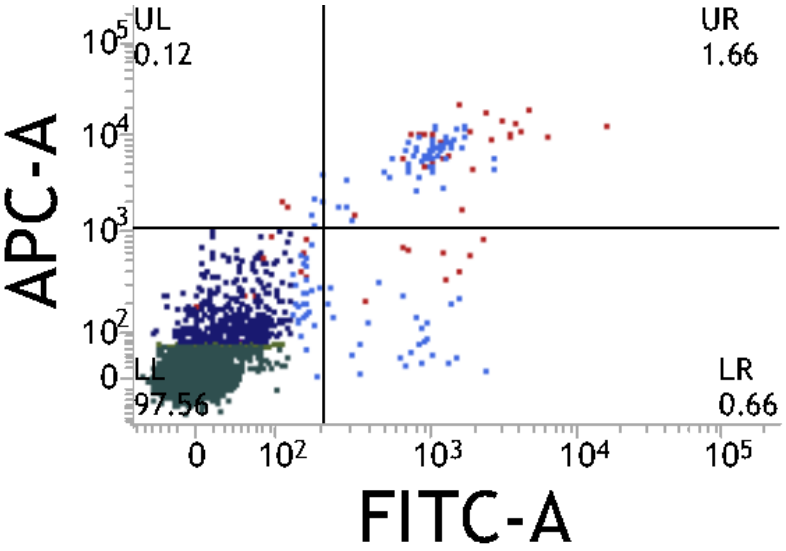
UL
0.12
UR
1.66
LL
97.56
LR
0.66
APC-A
FITC-A

E

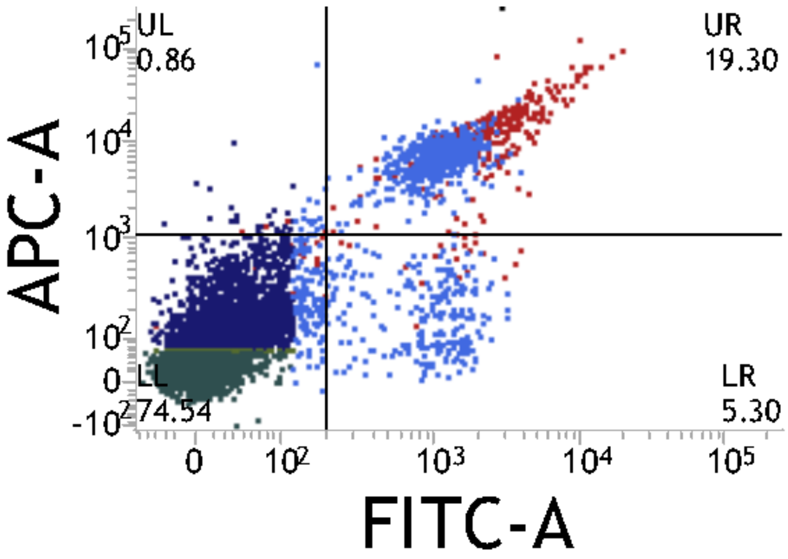
UL
0.86
UR
19.30
LL
74.54
LR
5.30
APC-A
FITC-A

F

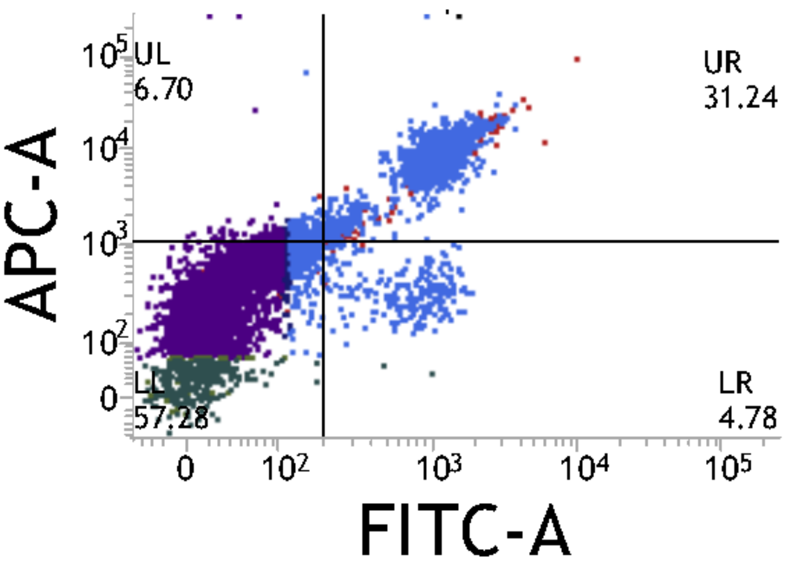
UL
6.70
UR
31.24
LL
57.28
LR
4.78
APC-A
FITC-A

G

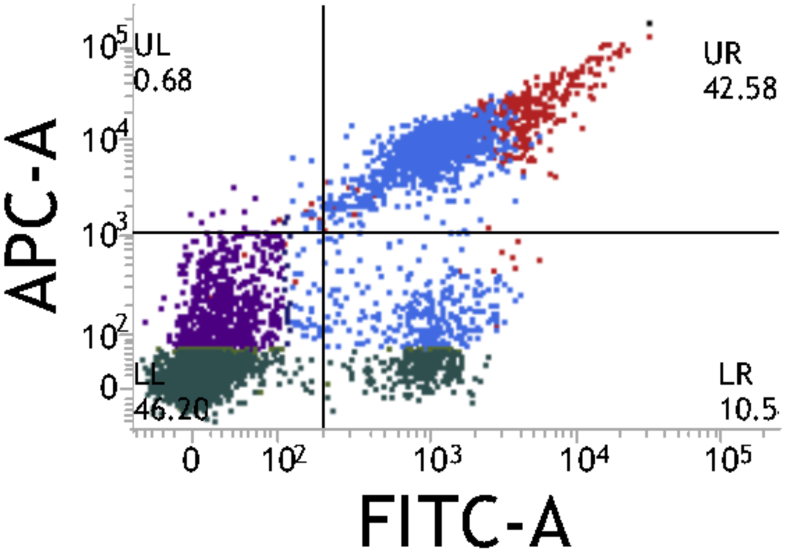
UL
0.68
UR
42.58
LL
46.20
LR
10.5
APC-A
FITC-A

H

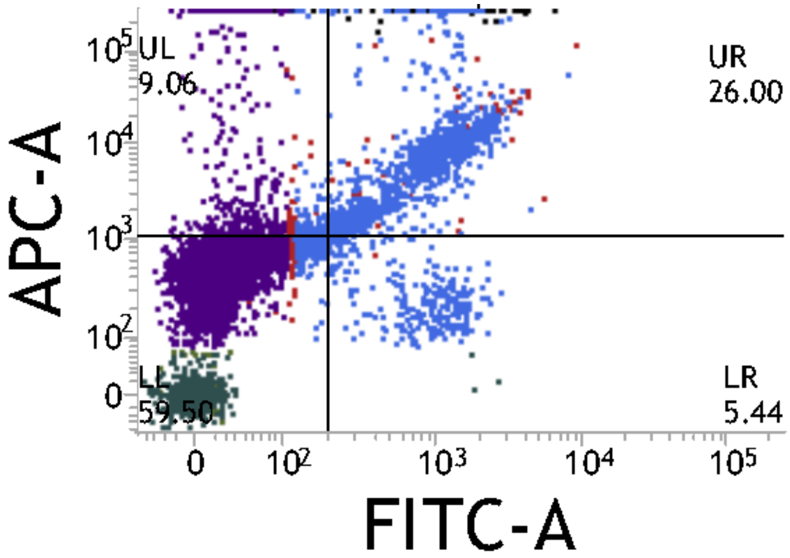
UL
9.06
UR
26.00
LL
59.50
LR
5.44
APC-A
FITC-A
0
10^2
10^3
10^4
10^5

I

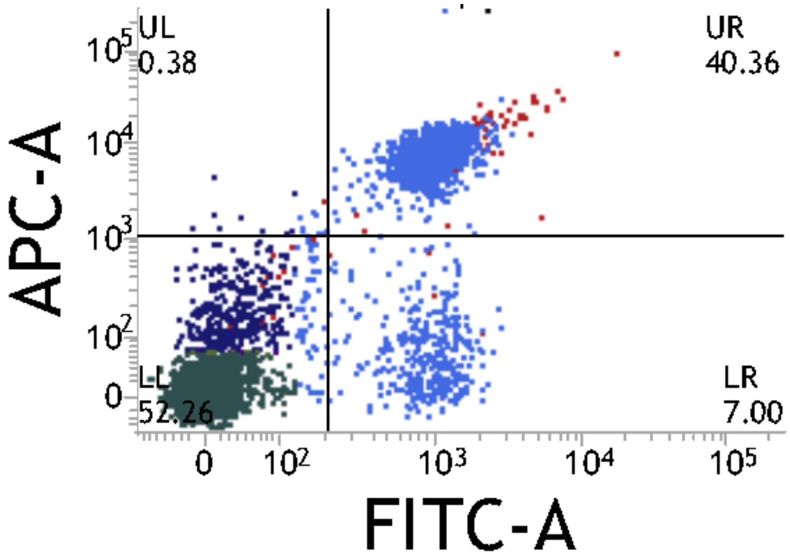
UL
0.38
UR
40.36
LL
52.26
LR
7.00
APC-A
FITC-A
0
10^2
10^3
10^4
10^5

Supplementary Figure 4 Representative flow cytometry plots showing percentage of cells positive for Ly6B-FITC and Ly6G-APC (markers for neutrophils) and Cd11b-FITC and Ly6C-APC (markers for monocytes) in control, MSU, MSU + Anti-CD44 antibody and MSU + Isotype Control (IC) antibody. **A)** Control, neutrophils; **B)** Control, monocytes; **C)** MSU, neutrophils; **D)** MSU, monocytes; **E)** MSU + Anti-CD44, neutrophils; **F)** MSU + Anti-CD44, monocytes; **G)** MSU + IC, neutrophils; and **H)** MSU + IC, monocytes.

A

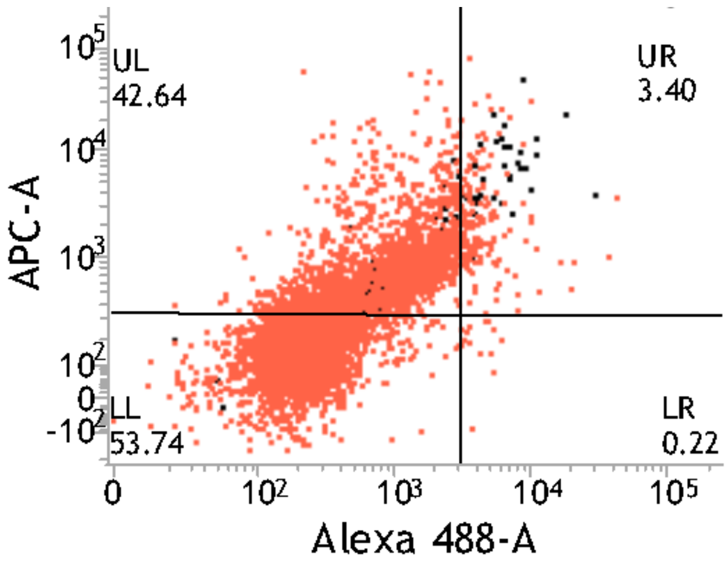

B

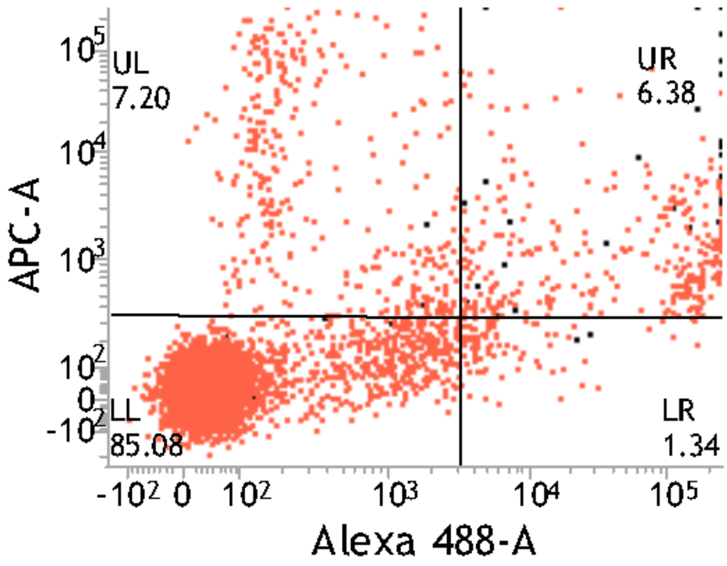

C

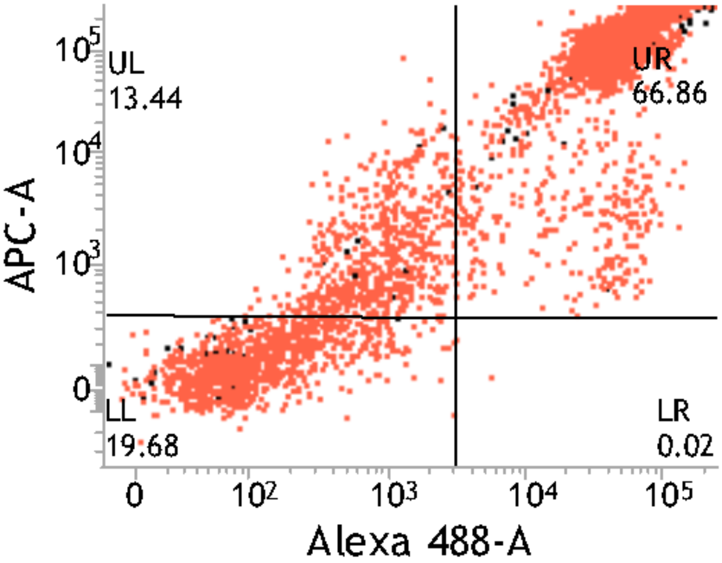
UL
13.44
UR
66.86
LL
19.68
LR
0.02
APC-A
Alexa 488-A

D

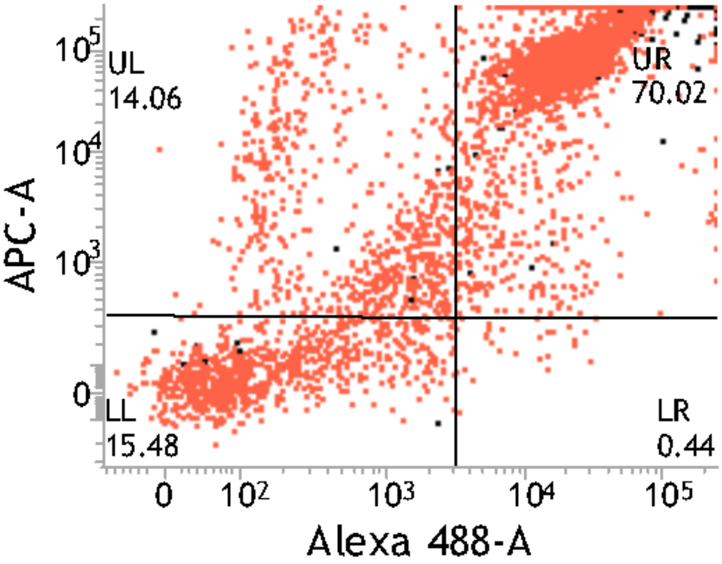
UL
14.06
UR
70.02
LL
15.48
LR
0.44
APC-A
Alexa 488-A

E

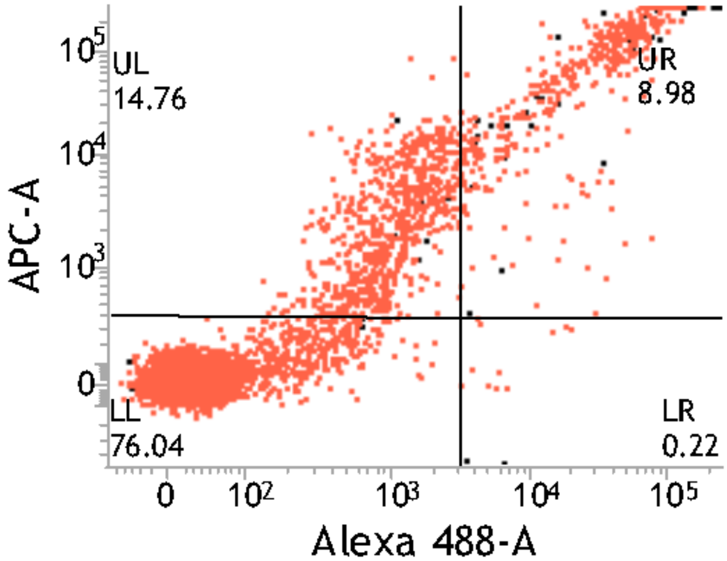
UL
14.76
UR
8.98
LL
76.04
LR
0.22
APC-A
Alexa 488-A

F

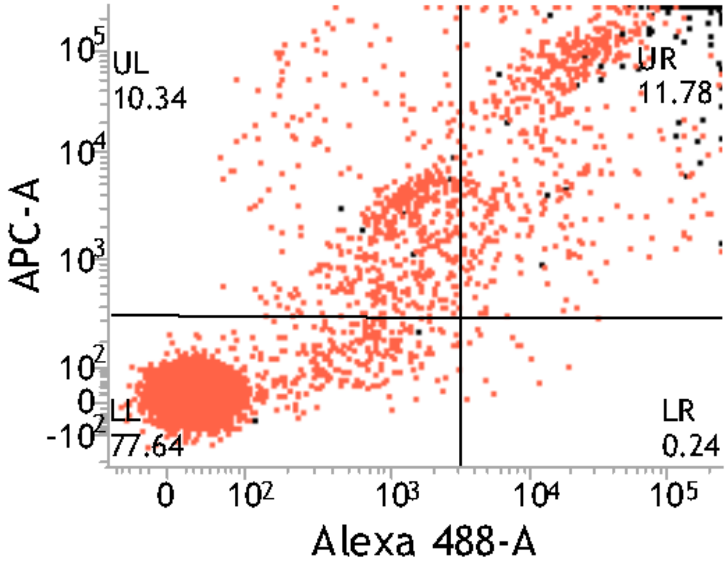
UL
10.34
UR
11.78
LL
77.64
LR
0.24
APC-A
Alexa 488-A

G

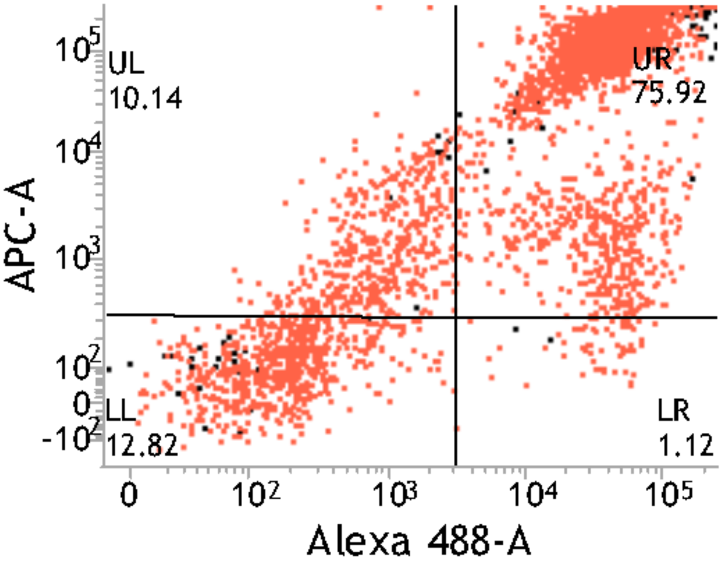
UL
10.14
UR
75.92
LL
12.82
LR
1.12
APC-A
Alexa 488-A

H

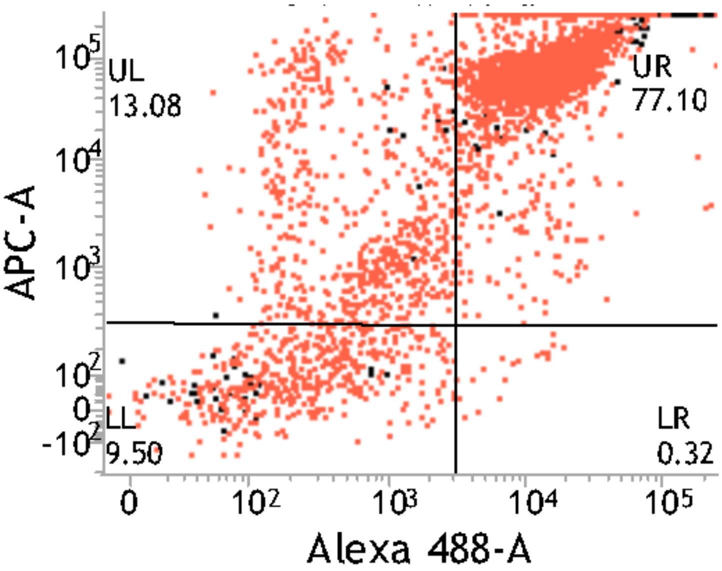
UL
13.08
UR
77.10
LL
9.50
LR
0.32
APC-A
Alexa 488-A
